# Combining Machine Learning and Molecular Simulations to Unlock Gas Separation Potentials of MOF Membranes and MOF/Polymer MMMs

**DOI:** 10.1021/acsami.2c08977

**Published:** 2022-07-12

**Authors:** Hilal Daglar, Seda Keskin

**Affiliations:** Department of Chemical and Biological Engineering, Koc University, Rumelifeneri Yolu, Sariyer, 34450 Istanbul, Turkey

**Keywords:** machine learning, mixed matrix membrane, permeability, selectivity, gas separation

## Abstract

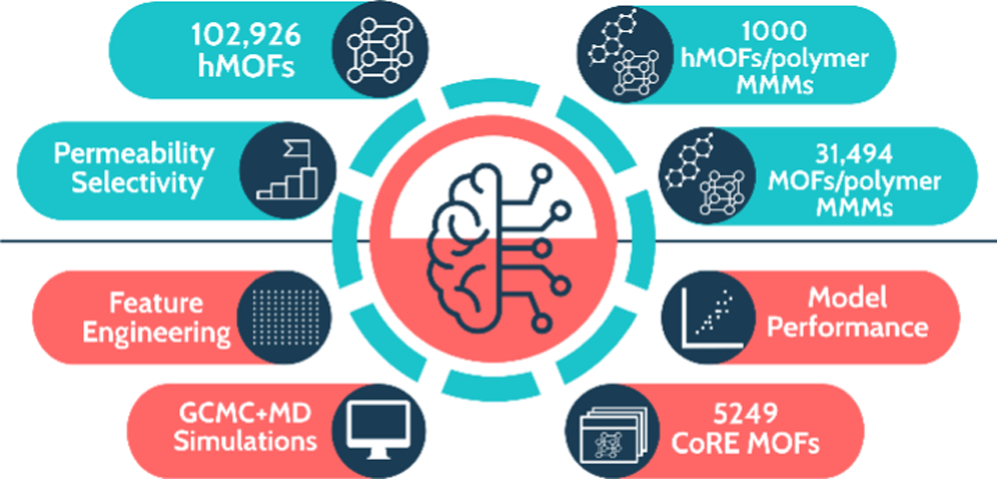

Due to the enormous increase in the number of metal-organic frameworks (MOFs), combining molecular simulations with machine learning (ML) would be a very useful approach for the accurate and rapid assessment of the separation performances of thousands of materials. In this work, we combined these two powerful approaches, molecular simulations and ML, to evaluate MOF membranes and MOF/polymer mixed matrix membranes (MMMs) for six different gas separations: He/H_2_, He/N_2_, He/CH_4_, H_2_/N_2_, H_2_/CH_4_, and N_2_/CH_4_. Single-component gas uptakes and diffusivities were computed by grand canonical Monte Carlo (GCMC) and molecular dynamics (MD) simulations, respectively, and these simulation results were used to assess gas permeabilities and selectivities of MOF membranes. Physical, chemical, and energetic features of MOFs were used as descriptors, and eight different ML models were developed to predict gas adsorption and diffusion properties of MOFs. Gas permeabilities and membrane selectivities of 5249 MOFs and 31,494 MOF/polymer MMMs were predicted using these ML models. To examine the transferability of the ML models, we also focused on computer-generated, hypothetical MOFs (hMOFs) and predicted the gas permeability and selectivity of 1000 hMOF/polymer MMMs. The ML models that we developed accurately predict the uptake and diffusion properties of He, H_2_, N_2_, and CH_4_ gases in MOFs and will significantly accelerate the assessment of separation performances of MOF membranes and MOF/polymer MMMs. These models will also be useful to direct the extensive experimental efforts and computationally demanding molecular simulations to the fabrication and analysis of membrane materials offering high performance for a target gas separation.

## 1 Introduction

Metal-organic frameworks (MOFs) have become a well-known class of materials to solve energy-related gas separation challenges due to their high porosities, large surface areas, and easy-to-modify structural properties.^1,2^ Due to the virtually unlimited combinations of metal parts and organic ligands, an enormous number of MOFs (>105,000) have been synthesized to date.^3^ MOFs have been widely investigated for gas storage and separation applications such as H_2_ storage, CH_4_ storage, CO_2_ capture, H_2_ purification, and separation of CO_2_ from natural gas and flue gas.^4−7^ Due to the environmental and economic advantages of membrane-based gas separations,^8^ MOFs have been studied as membrane materials.^9^ Experimental fabrication and testing of each MOF membrane for a target gas separation are not practical in terms of time and cost; thus, computational screening plays an important role in assessing the gas separation performances of a large number of MOFs to identify the top promising membranes.^10−12^ Several computational screening studies, which use molecular simulations to assess MOF membranes for various gas separations, CO_2_/N_2_, CO_2_/CH_4_, H_2_/CO_2_, O_2_/N_2_, and Xe/Kr, have been reported.^13−17^ However, performing computationally demanding grand canonical Monte Carlo (GCMC) and molecular dynamics (MD) simulations for several thousands of MOFs, analyzing and interpreting the very large amount of simulated data while keeping up with the fast progress of discovery of new MOFs are the current challenges in this field.

Machine learning (ML) is an excellent approach to analyzing a large amount of simulated material data since establishing structure–performance relations for MOFs can lead to the design and development of new MOF materials with better performances.^18^ In the last several years, ML algorithms have been used to study MOFs for various adsorption-based gas separations such as CO_2_ capture,^19−21^ H_2_O/(O_2_ + N_2_),^22^ H_2_S/CH_4_,^23^ propane/propylene,^24^ and Xe/Kr^25^ separations. On the other hand, ML has been used to study MOF membranes in a very limited number of studies due to the difficulty of generating gas permeability data using computationally demanding MD simulations. Zhou et al.^26^ used different ML algorithms to predict the D_2_/H_2_ selectivity of MOF membranes at infinite dilution, 77 K, and found that the D_2_/H_2_ membrane selectivity of the best MOFs is one order of magnitude higher than those previously reported in the literature. Qiao et al.^27^ used the ML approach to compute the relative importance of MOF features on the predicted membrane selectivities and showed that porosity and the largest cavity diameter (LCD) have high importance. Zhong et al.^28^ developed an ML model to predict *i*-C_4_H_8_ permeability and *i*-C_4_H_8_/C_4_H_6_ selectivity of 601 covalent organic framework (COF) membranes at 1 bar, 298 K, and showed that porosity and pore limiting diameter (PLD) are key factors controlling the selectivity and permeability of COF membranes. Bai et al.^29^ recently developed eight different ML algorithms to predict H_2_ permeability, H_2_/CH_4_ membrane selectivity, and trade-off multiple selectivity and permeability (TMSP) of MOFs and showed that two ML models are the most suitable ones for predicting the H_2_ separation performances of MOFs. In our recent study, ML models were trained to predict O_2_/N_2_ adsorption, diffusion, and membrane selectivities of 5632 MOFs and 137,953 hypothetical MOFs (hMOFs) at 1 bar, 298 K, to identify the hMOFs with high O_2_/N_2_ selectivity.^30^

Compared to MOF membranes, a much larger variety of MOF/polymer mixed matrix membranes (MMMs) have been fabricated and the incorporation of MOFs as fillers into polymers to generate MMMs has been shown to improve the gas permeability and/or selectivity of the pure polymer in several experimental and computational studies.^31,32^ The gas adsorption and diffusion data of MOFs obtained from GCMC and MD simulations have been used to estimate the gas permeability of the MOF/polymer MMMs in computational studies,^14,33^ and this approach has been shown to provide accurate predictions for CO_2_/N_2_,^32^ CO_2_/CH_4_,^34^ and H_2_/N_2_^35^ separation performances of MOF/polymer MMMs. Although a large number of MOF/polymer MMM studies exist in the literature, no ML study has been reported to predict the gas permeabilities of these MMMs to date.

In this study, we combined the ML and large-scale molecular simulation approaches to assess the potential of both MOF membranes and MOF/polymer MMMs for six different gas separations, He/H_2_, He/CH_4_, He/N_2_, H_2_/CH_4_, H_2_/N_2_, and N_2_/CH_4_. We first performed GCMC and MD simulations to obtain the adsorption and diffusion properties of He, H_2_, N_2_, and CH_4_ gases for the total of 5249 MOFs at 1 bar, 298 K. We then developed ML models that can accurately predict the uptake and diffusivities of the gases in MOFs. By using the ML-predicted gas uptake and diffusivity, we calculated the gas permeabilities and selectivities of the total of 5249 MOF membranes and 31,494 different MOF/polymer MMMs composed of six polymers for six different gas separations. We finally investigated the transferability of our ML models to unseen computer-generated, hMOF data set for predicting the gas permeability and selectivity of 1000 hMOF/polymer MMMs composed of 500 hMOFs and 2 polymers. The ML models that we developed in this work will be very useful to accurately and rapidly predict gas permeabilities and selectivities of MOF membranes and MOF/polymer MMMs without performing computationally demanding molecular simulations. These predictions will be useful to accelerate both the identification and fabrication of the best-performing MOF membranes and MOF/polymer MMMs for various types of gas separations. The ML models that we developed also revealed the most important MOF features for high gas permeabilities and selectivities so that they will shed light on the design of new high-performing membrane materials that have not been fabricated yet.

## 2 Methods

**Figure 1:**
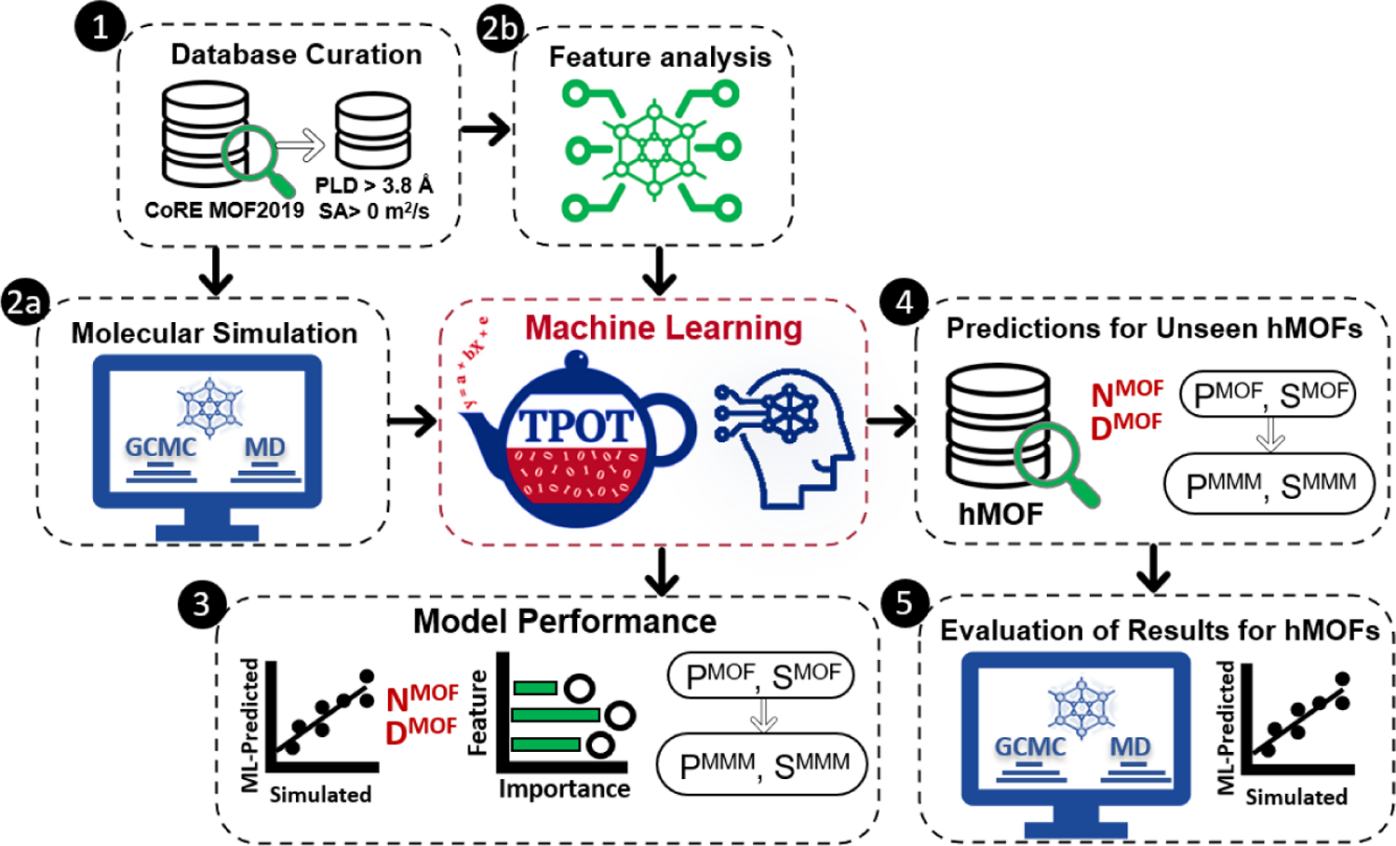
Computational workflow of this study: (1) selection of the MOFs based on the pore sizes and accessible surface areas; (2a) performing molecular simulations to obtain the adsorption and diffusion data of He, H_2_, CH_4_, and N_2_ in MOFs; (2b) analyzing features and determining the physical and chemical descriptors of MOFs; (3) comparing the ML-predicted uptake, diffusion, and permeability of gases with the simulated results of MOF membranes and MOF/polymer MMMs; (4) predicting the uptake, diffusion, and permeability of gases for the unseen hMOF data set using the ML models generated; and (5) evaluating the accuracy of ML models for the unseen hMOFs by comparing ML-predicted data with the simulated data of unseen hMOF.

Our computational methodology combining molecular simulations and ML to examine gas separation performances of MOF-based MMMs is illustrated in Figure 1. We first filtered the MOF database by setting two criteria related to pore size and surface area of MOFs to enable the adsorption of gases in the MOFs’ pores (step 1). Gas adsorption and diffusion in MOFs were then investigated by performing molecular simulations, GCMC and MD, respectively (step 2a), which were used as target data in our ML models. The physical, chemical, and energetic features of MOFs such as pore size, pore geometry, atom types, metallic percentage, and heat of adsorption of gases in MOFs were analyzed (step 2b), and these features were used as input variables for training ML models to predict the target data, gas uptake, and diffusivity in MOFs. Using input variables and target data, we trained and developed ML models. ML-predicted gas adsorption and diffusion data were compared with the simulated data of MOFs to determine the accuracy of these ML models (step 3).

We then obtained gas permeability and selectivity of MOF membranes and MOF-based MMMs using the gas adsorption and diffusion data computed from molecular simulations and predicted from ML models (step 3). The ML models were finally used to predict the gas adsorption and diffusion properties of unseen hypothetical MOFs (hMOF) (step 4) by repeating the same steps (steps 1–3) for them. Molecular simulation results were compared with the ML predictions for hMOF membranes and hMOF/polymer MMMs. More details about the data refinement, molecular simulations, and generation of ML models are given below.

### 2.1 Curation of the MOF Data Set

In this study, we used the most recent collection of experimentally synthesized MOF database (CoRE MOF 2019), which consists of 12,020 materials.^36^ As shown in Figure 1, we narrowed down the CoRE MOF data set by focusing on the MOFs with PLD > 3.8 Å and accessible surface area (SA) >0 m^2^/g so that all gas molecules that we studied (He, H_2_, N_2_, and CH_4_) can pass through the MOFs’ pores. Since the output of GCMC simulations (loading and positions of the gas molecules in MOFs) was used as the initial states of MD simulations, we only studied the MOFs for which GCMC simulations resulted in at least one molecule of adsorbed gas per structure. After MD simulations, we only considered the MOFs exhibiting gas self-diffusivities >10^–8^ cm^2^/s, the limit to accurately characterize molecular diffusion in MOFs using MD.^37^ In training ML models, we defined the cutoff threshold values for uptakes and diffusivities of He, H_2_, N_2_, and CH_4_, as shown in Table S1, to refine the data and increase the accuracy of ML models. Using these threshold values, a small number of MOFs (0.2, 0.6, 0.8, and 1.7% of all MOFs for He, N_2_, CH_4_, and H_2_, respectively) was identified as outliers and eliminated. For the ML models developed for He and H_2_ diffusion, we calculated the difference between the simulated and ML-predicted diffusivities and computed the standard deviation for each MOF. If this difference was greater than double of the standard deviations of the training data for any MOF, then this MOF was not used in the training of models. We finally note that the MOF set used to train ML models for adsorption and diffusion was identical for a given gas. Having gone through these steps, we ended up with 677 MOFs for training ML models for He, 2715 MOFs for H_2_, 5215 MOFs for CH_4_, and 5224 MOFs for N_2_.

### 2.2 Molecular Simulations and Membrane Calculations

We computed gas uptakes (N*_i_*) and self-diffusivities (D*_i_*) of He, H_2_, N_2_, and CH_4_ by performing GCMC and MD simulations, respectively, at 1 bar, 298 K. All simulations were performed using RASPA software.^38^ Dispersion interactions between MOF–gas and gas–gas were described with Lennard-Jones 12-6 (LJ) potentials. The universal force field (UFF)^39^ parameters were used for the framework atoms. While CH_4_,^40^ H_2_,^41^ and He^40^ were modeled as single, spherical, and nonpolar atoms, N_2_ was modeled as three-site molecules: two N atoms and a dummy atom as the center of mass.^42^ N_2_ has quadrupole moments for which electrostatic interactions between the gas and the MOFs were considered. The charge equilibration method (Qeq)^43^ as implemented in RASPA was used to estimate the partial atomic charges of MOFs. The Ewald summation was used to calculate the long-range electrostatic interactions.^44^ The potential parameters of gases are listed in Table S2. In GCMC simulations, we used 2 × 10^4^ cycles for initialization and another 2 × 10^4^ cycles for taking the ensemble averages. In MD simulations, NVT ensemble was used, where the step size and total simulation time were 1 fs and 5 ns at 298 K, respectively. We run MD simulations for 5 × 10^6^ cycles, using 10^3^ cycles for initialization and 10^4^ cycles for the equilibration of each MOF. More details of simulations can be seen in our previous works.^14,33^ By using simulated gas adsorption and diffusion data, gas permeabilities of MOFs were calculated using *P*_i_ = *c_i_× D_i_*/*f*_*i*_, where c*_i_*, *D_i_*, and *f_i_* represent the adsorbed concentration, self-diffusivity, and feed side pressure of gas *i*, respectively. The feed (permeate) side of the membrane was assumed to be at 1 bar (under vacuum).^45^ Then, ideal membrane selectivities were calculated as the ratio of single-component gas permeabilities, *S*_*i*/*j*_^mem^ = *P_i_*/*P_j_*.

MOF-based MMMs were studied for six different separations, He/H_2_, He/N_2_, He/CH_4_, H_2_/N_2_, H_2_/CH_4_, and N_2_/CH_4_. For each separation, we selected at least three polymers representing membranes with high, medium, and low gas permeabilities, which defined Robeson’s upper bound.^46^ Experimentally reported gas permeabilities of these polymers are listed in Table S3. To predict the gas permeabilities of the MOF-based MMMs, we used the Maxwell model^47^ since it was previously shown that the simulated gas permeability of MOF-based MMMs calculated by this model agrees well with the experimental data.^14,33^ Maxwell model uses simulated gas permeability data of MOFs and experimentally measured gas permeability data of polymers to compute the gas permeability of MOF/polymer MMM as follows, *P*_i_^MMM^ = *P*_i_^P^ × . Here, *P*_i_^MMM^, *P*_i_^MOF^, and *P*_i_^P^ represent the gas permeability of MMM, MOF, and polymer, respectively. ϕ is the volume fraction of MOF fillers in the polymer and was used as 0.2 throughout this study. We calculated the He permeabilities of 2031 MMMs, H_2_ permeabilities of 10,860 MMMs, CH_4_ permeabilities of 26,075 MMMs, and N_2_ permeabilities of 31,344 MOF-based MMMs. The ratio of gas permeabilities was used to compute the selectivities of MMMs, *S*_*i*/*j*_^MMM^ = *P*_i_^MMM^/*P*_j_^MMM^.

### 2.3 Feature Analysis of MOFs

The ML models aim to establish the relations between MOF descriptors and the target data, which are the gas uptake and diffusivity data of MOFs at 1 bar, 298 K. Ideally, descriptors should be easy to obtain/calculate and have low dimensionality and correlation with the target data to some extent. We extracted 20 different features as potential descriptors in Table 1. LCD, PLD, and their ratios (LCD/PLD) were shown to affect the adsorption and diffusion of gases in MOFs.^15,32,48,49^ We also considered the features of the pore geometry such as pore volume, porosity, density, and SA, which are commonly used in ML studies.^50−53^

**Table 1:** Descriptors Used to Construct a Feature Vector for ML Models

group^a^	feature (unit)	symbol
A	largest cavity diameter (Å)	LCD
	pore limiting diameter (Å)	PLD
	pore size ratio	LCD/PLD
B	density (g/cm^3^)	ρ
	pore volume (cm^3^/g)	PV
	porosity	φ
	surface area (m^2^/g)	SA
C	carbon percentage	C%
	hydrogen percentage	H%
	nitrogen percentage	N%
	oxygen percentage	O%
	halogen (Br, Cl, F, I) percentage	halogen%
	metalloid (As, B, Ge, Te, Sb, Si) percentage	metalloid%
	ametal (Se, S, P) percentage	ametal%
	metal percentage	metal%
D	total degree of unsaturation	TDU
	degree of unsaturation	DU
	metallic percentage (#of metal/#of C atoms)	MP
	oxygen-to-metal ratio	O-to-M
E	heat of adsorption (kJ/mol)	*Q*_st_^0^

a The features are separated into five groups. A–E represent features of the pore size, pore geometry, atom types, and chemical and energy-based descriptors, respectively.

To further improve the predicting power of ML models, we also used the atom types in the frameworks, which is the number of specified elements divided by the number of total atoms in a unit cell of MOF multiplied by 100, such as C%, H%, and metal%. Degree of unsaturation (DU), which indicates the total number of π bonds and rings, total degree of unsaturation (TDU), metallic percentage (MP), and oxygen-to-metal ratio (O-to-M) are essential chemical descriptors describing the molecular structures.^54^ While the descriptors related to pore size and geometry such as PLD, LCD, and porosity were calculated using Zeo++ software,^55^ atom type and chemical descriptors were extracted from the crystallographic information files (CIFs) of MOFs taken from the CoRE MOF database. A nitrogen probe with a radius of 1.86 Å and 2 × 10^3^ trials were used for the surface area calculations. Geometric pore volumes were computed using a probe radius of 0 Å and 5 × 10^4^ trials. We finally used the isosteric heat of adsorption values (*Q*_st_^0^) of gases computed at infinite dilution, 298 K, using the Widom insertion method as the energy descriptor in ML models developed for N_2_ adsorption and diffusion.^37^ Details for computing *Q*_st_^0^ using molecular simulations can be found in our previous work.^14^

The Pearson correlation coefficient (*r*) was used to determine the feature correlations, which can be expressed as , where *x* and *y* are the features, and *x̅* and *y̅* are the means of *x* and *y*. If the two descriptors are strongly correlated, it can cause problems such as multicollinearity and overtraining of ML models.^56^ To avoid these, we computed the *r* values between each descriptor and removed the one having a strong correlation (*r* > 0.90).

### 2.4 Machine Learning

We used the tree-based pipeline optimization tool (TPOT)^57^ in auto-machine learning^58^ to efficiently select the best algorithm and optimize the model parameters. TPOT is based on the evolutionary algorithm (EA) optimization and includes three steps of ML: feature engineering, model generation, and model evaluation. In TPOT, a random principal singular value decomposition variant called randomized principal component analysis (PCA)^59^ is used for feature extraction. Comparison of a CH_4_ working capacity of 403,959 hypothetical COFs predicted using the algorithms defined by TPOT and traditional ML models such as decision tree (DT), random forest (RF), and support vector machine (SVM) showed that the accuracy of ML predictions obtained from TPOT is higher than those of traditional ML models.^56^ For the model selection in TPOT, the regression algorithms in the scikit-learn toolkit^59^ were used. A stratified sampling method was implemented to keep the feature distribution in training and test data as consistent as possible. The data was split into two sets, 80% as a training set and 20% as a test set. We also used a fivefold cross-validation to avoid overfitting. TPOT parameters listed in a table were provided on GitHub (https://github.com/hdaglar/MOF-basedMMMs_ML). We compared the range of descriptors in the training and test sets for He, H_2_, N_2_, and CH_4_ in Figures S1 and S2 and showed that the feature distribution in the training and test sets is similar for each gas species. Results also highlighted that the MOFs in the training set are representative of the entire MOF set, providing more accurate predictions for the test set with similar characteristics.

To evaluate the model accuracy, we used the coefficient of determination (*R*^2^), mean absolute error (MAE), and root-mean-square error (RMSE) as follows

, ,  Here, *M* represents the number of samples, *y* and *ŷ* represent the simulated (true) value and predicted value, respectively, and *y̅* denotes the average of the simulated value by the model. As RMSE and MAE increase, the accuracy of models decreases. We also used the Spearman rank correlation coefficient (SRCC) to calculate the ranking correlation between simulated and ML-predicted data using , where *D* is the difference between paired ranks and *n* is the number of observations. SRCC is an important tool to understand how well the two rankings agree. As the value of SRCC increases, the similarity between the two rankings and the accuracy of models increase. Based on RMSE, MAE, and *R*^2^, the results of the ML algorithms with their optimized parameters are presented in Table S4. The best ML algorithms for predicting the adsorption and diffusion properties of He, H_2_, CH_4_, and N_2_ in MOFs were found as LassoLarsCV, Extra Trees Regressor, Gradient Boosting Regressor, and Random Forest Regressor. The last three are tree-based ensemble methods, while LassoLarsCV is a regulated linear regression model implemented using the least angle regression (Lars) algorithm and cross-validation (CV). We note that these models (Lasso,^7^ Random Forest,^24,30^ Gradient Boosting^20^) have been commonly used to train ML models for MOFs.

After developing the ML models for predicting the gas separation performances of the MOF membranes and MOF/polymer MMMs, we focused on the hypothetical MOF (hMOF) database,^60^ which includes 137,593 computer-generated materials to test the transferability of our ML models to a different material database. We eliminated the hMOFs with nonaccessible SA and PLD < 3.8 Å and ended up with 102,926 hMOFs. Performing molecular simulations for that many materials is computationally very expensive. Therefore, we ranked 102,926 hMOFs based on their LCDs and created a representative subset composed of 500 materials, which involve 1st hMOF and every 205th hMOF thereafter. Figure S3 shows that the ranges of all features of our representative hMOF set (500 hMOFs) are similar to those of the complete hMOF set (102,926 hMOFs). Then, we predicted He, H_2_, N_2_, and CH_4_ uptakes and diffusivities in 500 hMOFs using the ML models that we developed for MOFs. GCMC and MD simulations were then performed to compute He, H_2_, N_2_, and CH_4_ adsorption and diffusion in 500 hMOFs following the simulation methods described in Section 2. ML-predicted (simulated) gas permeabilities of hMOFs were obtained using the ML-predicted (simulated) gas uptakes and diffusivities. Finally, we compared the simulated and ML-predicted gas permeabilities and selectivities of 1000 hMOF/polymer MMMs composed of 2 polymers and 500 hMOFs.

## 3 Results and Discussion

### 3.1 Feature Correlation and Univariate Analysis

**Figure 2:**
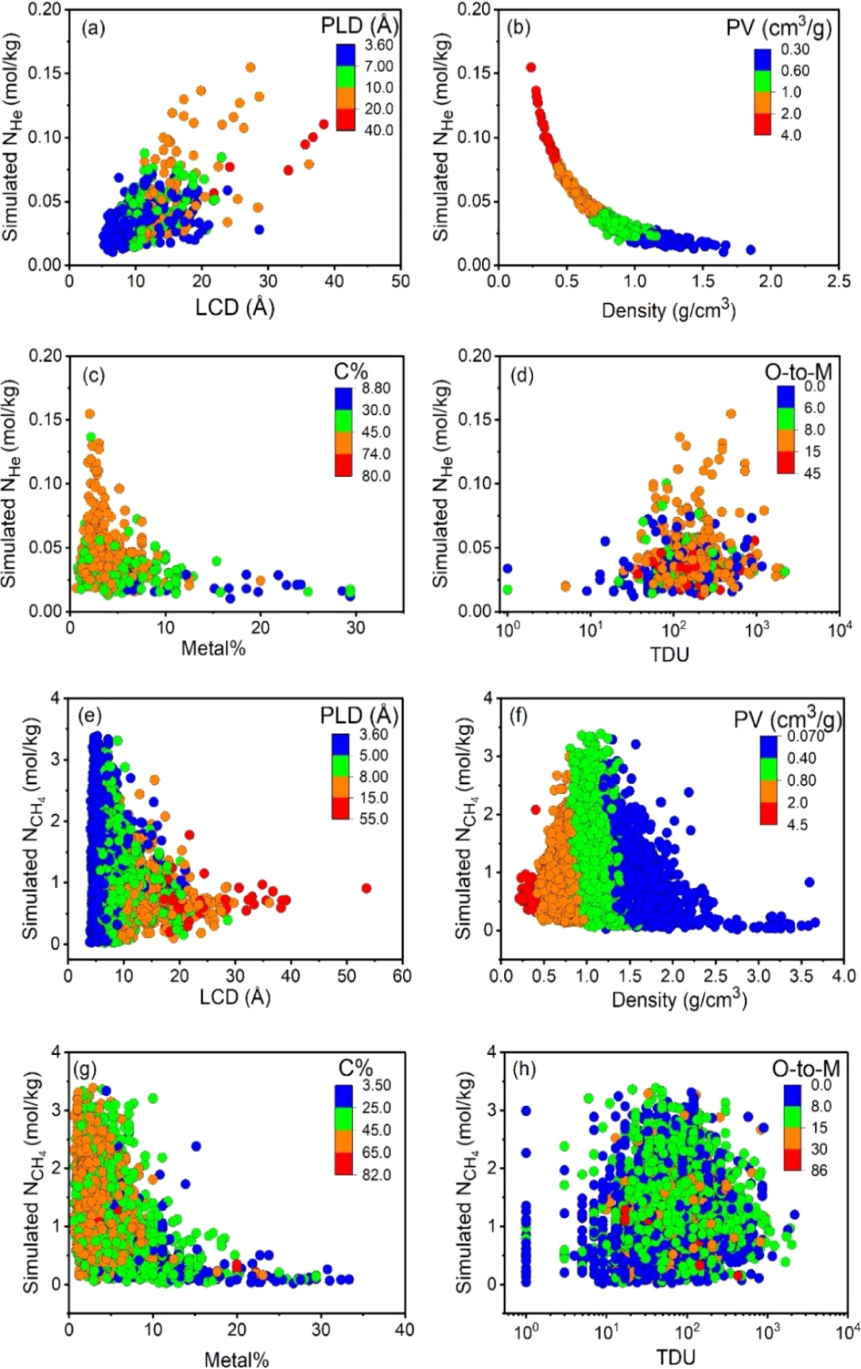
Effect of features on gas adsorption: simulated He uptakes in 677 MOFs as a function of (a) pore size (LCD, PLD), (b) pore geometry (density, pore volume), (c) atom types (C%, metal%), and (d) chemical descriptors (O-to-M, TDU). Simulated CH_4_ uptakes in 5215 MOFs as a function of (e) pore size (LCD, PLD), (f) pore geometry (density, pore volume), (g) atom types (C%, metal%), and (h) chemical descriptors (O-to-M, TDU).

After the descriptors were determined, relations between these descriptors and the simulated gas adsorption and diffusion data of MOFs were examined. We focused on two features in each group of the descriptors: LCD and PLD for the pore size, pore volume, and density for the pore geometry, C% and metal% for the atom types, and O-to-M and TDU for the chemical descriptors. Figure 2 illustrates the correlations between these features and uptakes for He and CH_4_. Figure 2a shows that the He uptake in MOFs generally increases as the LCDs and PLDs expand. Not surprisingly, the MOF density and He uptake have an inverse relationship, implying that high pore volume generally leads to high He uptake, as shown in Figure 2b. Figure 2c,d shows that He adsorption is typically favored in the MOFs having high C% and low metal%. Figure 2e represents that the MOFs with narrow pore sizes are favorable for high CH_4_ uptake. For many MOFs, CH_4_ uptake increases as the framework density increases up to 1.5 g/cm^3^ and generally decreases in denser MOFs (>1.5 g/cm^3^), as shown in Figure 2f. While CH_4_ uptake generally increases as the C% increases, there is an inverse relation between the metal% and CH_4_ uptake, as shown in Figure 2g. There is almost no observable correlation between the CH_4_ uptake and chemical descriptors in Figure 2h. We observed similar results for H_2_ and N_2_ uptakes, as shown in Figure S4. Overall, some features correlate with the gas uptake of MOFs, but many exceptions exist complicating the explanation of the structure–performance relations.

Figure S5 represents the relations between He and CH_4_ diffusion in MOFs and material features. He self-diffusivity in MOFs increases as PLD and LCD increase in Figure S5a. While there is a linear correlation between the pore volume and He diffusion, an inverse relation between density and diffusivity is observed in Figure S5b. Atom types and chemical descriptors weakly correlate with He diffusivity in Figure S5c,d. High CH_4_ diffusion is generally observed in MOFs having large PLD, large LCD, low density, high pore volume, low-medium C%, and high metal%, as shown in Figure S5e–g.

There is almost no correlation between the chemical descriptors and CH_4_ diffusivity, as shown in Figure S5h. Similar results were obtained for self-diffusivities of H_2_ and N_2_, as shown in Figure S6. We inferred that, compared to the gas uptake, there is a weaker relation between MOF features and gas diffusivities since the movement of the gas molecules through the MOFs’ pores is generally more complicated than the adsorption of gas molecules in the pores of MOFs.

### 3.2 Predictions of ML Models for MOF Membranes

Considering the results of the previous section, we employed the pore size, pore geometry, chemical descriptors, and atom types (shown in Table 1) to train eight ML models to describe the uptakes and diffusivities of He, H_2_, N_2_, and CH_4_ in MOFs. Figure S7 shows the heatmap with the Pearson correlations across different features of MOFs. Although there are strong correlations between some features such as pore volume and porosity (*r*: 0.82), LCD and PLD (*r*: 0.77), no pair of features is overly correlated (*r* > 0.9), suggesting that all features can be used as input variables while training the ML models.^56^ Therefore, we considered all of the features given in Table 1 to investigate how the descriptor selection affects the accuracy of ML models.

**Figure 3:**
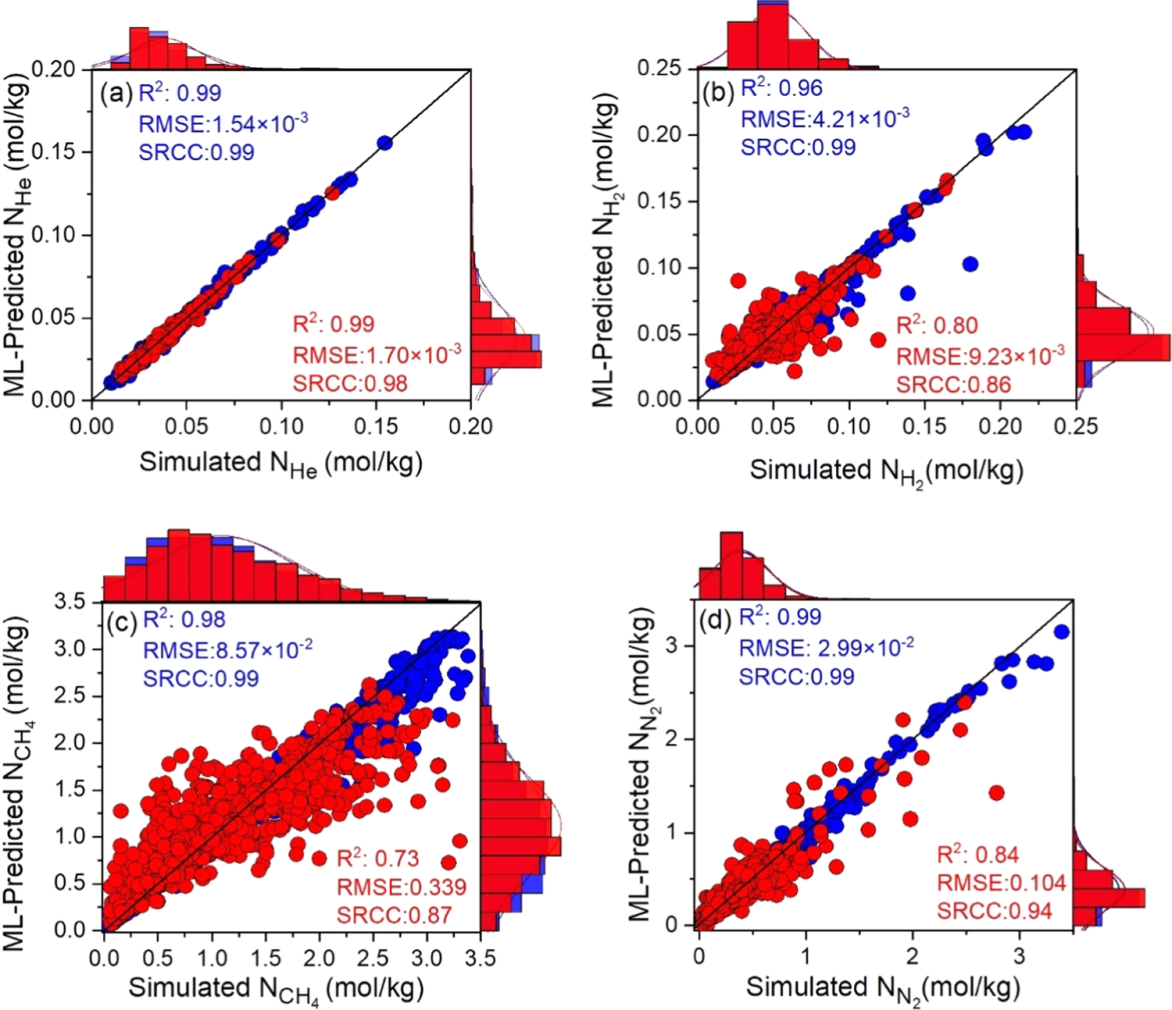
Comparison of the ML-predicted adsorption of (a) He, (b) H_2_, (c) CH_4_, and (d) N_2_ in MOFs with the simulation results. Blue (red) symbols represent the training (test) data.

Table 2 lists *R*^2^, MAE, RMSE, and SRCC of the training and test sets based on the feature groups. While our simplest ML model was established using only pore size (group A), other features were added to build extended, more predictive/accurate models such as A+B, A+B+C, A+B+C+D, and A+B+C+D+E. For example, when pore size and pore geometry (A+B) were used to predict the CH_4_ uptake in MOFs, *R*^2^ of the test set was computed as 0.6. When atom types and chemical descriptors were added to the feature list, *R*^2^ of the test set increased to 0.73. This shows the supportive effect of the atom types and the chemical descriptors in multivariate analysis, while they have almost no correlation with the gas uptake and/or diffusivity in univariate analysis, as previously shown in Figure 3. Table 2 also shows that pore size and pore geometry are the dominant features determining the accuracy of ML models for the gas uptake and diffusivity predictions. Incorporating the atom types and chemical descriptors into the ML models improved the accuracy of predictions only marginally. There can be slightly different trends (increase or decrease) in the calculated SRCC and *R*^2^ values of the training and test sets given in Table 2, which can be considered acceptable. The most pronounced change was observed for the H_2_ uptake model where SRCC and *R*^2^ values were decreasing from 0.999 to 0.986 and from 0.999 to 0.962 in the training set, while these values were increasing from 0.58 to 0.86 and from 0.38 to 0.80 in the test set, respectively. This might be due to the overfitting in the ML model using only A group of descriptors for H_2_ uptake. As shown in Table 2, when we used the A+B+C+D group of descriptors, *R*^2^ and SRCC of ML models for N_2_ uptake and diffusivity are not as high as those obtained for other gases. Therefore, we also included *Q*_st_^0^ in ML models for N_2_ uptake and diffusivity to improve the accuracy. We note that since experimental measurements and molecular simulations to determine *Q*_st_^0^ require more time and more inputs compared to other structural properties that we used, we did not use *Q*_st_^0^ in ML models for He, H_2_, and CH_4_ uptakes and diffusivities. Based on the analysis presented in Table 2, we used A+B+C+D (A+B+C+D+E) descriptor groups to train the ML models for predicting the uptake and diffusivity of He, H_2_, and CH_4_ (N_2_) in MOFs.

**Table 2:** Selection of Descriptor Groups for ML Models

	training set				test set			
	RMSE	MAE	SRCC	*R*^**2**^	RMSE	MAE	SRCC	*R*^2^
Descriptor Groups	He Uptake							
A	1.12 × 10^–2^	7.35 × 10^–3^	0.816	0.688	1.12 × 10^–2^	8.32 × 10^–3^	0.711	0.56
A+B	2.06 × 10^–3^	1.68 × 10^–3^	0.985	0.989	2.20 × 10^–3^	1.78 × 10^–3^	0.979	0.98
A+B+C	1.55 × 10^–3^	1.20 × 10^–3^	0.992	0.994	1.75 × 10^–3^	1.34 × 10^–3^	0.984	0.99
A+B+C+D	1.54 × 10^–3^	1.18 × 10^–3^	0.992	0.994	1.70 × 10^–3^	1.30 × 10^–3^	0.984	0.99
	He Diffusion							
A	4.00 × 10^–4^	3.11 × 10^–4^	0.859	0.751	6.06 × 10^–4^	4.79 × 10^–4^	0.592	0.41
A+B	3.53 × 10^–4^	2.85 × 10^–4^	0.87	0.805	4.81 × 10^–4^	4.05 × 10^–4^	0.719	0.63
A+B+C	3.20 × 10^–4^	2.56 × 10^–4^	0.902	0.84	4.63 × 10^–4^	3.89 × 10^–4^	0.758	0.64
A+B+C+D	3.29 × 10^–4^	2.63 × 10^–4^	0.894	0.831	4.76 × 10^–4^	3.90 × 10^–4^	0.747	0.65
	H_2_ Uptake							
A	1.0 × 10^–6^	1.0 × 10^–6^	0.999	0.999	1.65 × 10^–2^	1.20 × 10^–2^	0.576	0.38
A+B	4.57 × 10^–3^	2.63 × 10^–3^	0.976	0.954	1.03 × 10^–2^	6.56 × 10^–3^	0.818	0.75
A+B+C	2.67 × 10^–3^	1.26 × 10^–3^	0.993	0.985	9.71 × 10^–3^	5.82 × 10^–3^	0.846	0.78
A+B+C+D	4.21 × 10^–3^	2.03 × 10^–3^	0.986	0.962	9.23 × 10^–3^	5.45 × 10^–3^	0.862	0.80
	H_2_ Diffusion							
A	5.62 × 10^–4^	4.43 × 10^–4^	0.602	0.499	6.35 × 10^–4^	4.94 × 10^–4^	0.542	0.35
A+B	2.26 × 10^–4^	1.71 × 10^–4^	0.952	0.919	4.73 × 10^–4^	3.67 × 10^–4^	0.734	0.64
A+B+C	2.50 × 10^–4^	1.99 × 10^–4^	0.936	0.901	4.41 × 10^–4^	3.47 × 10^–4^	0.773	0.69
A+B+C+D	1.79 × 10^–4^	1.35 × 10^–4^	0.973	0.951	4.40 × 10^–4^	3.43 × 10^–4^	0.768	0.70
	CH_4_ Uptake							
A	5.25 × 10^–1^	4.04 × 10^–1^	0.587	0.322	6.03 × 10^–1^	4.68 × 10^–1^	0.39	0.14
A+B	2.34 × 10^–1^	1.60 × 10^–1^	0.94	0.865	4.12 × 10^–1^	2.86 × 10^–1^	0.792	0.60
A+B+C	4.67 × 10^–2^	2.75 × 10^–2^	0.998	0.995	3.38 × 10^–1^	2.11 × 10^–1^	0.872	0.72
A+B+C+D	8.57 × 10^–2^	4.79 × 10^–2^	0.995	0.981	3.39 × 10^–1^	2.12 × 10^–1^	0.874	0.73
	CH_4_ Diffusion							
A	9.17 × 10^–5^	6.19 × 10^–5^	0.793	0.59	1.17 × 10^–4^	7.97 × 10^–5^	0.62	0.31
A+B	3.56 × 10^–5^	2.11 × 10^–5^	0.974	0.938	7.46 × 10^–5^	4.66 × 10^–5^	0.861	0.72
A+B+C	1.22 × 10^–5^	6.76 × 10^–6^	0.997	0.993	6.72 × 10^–5^	4.10 × 10^–5^	0.889	0.77
A+B+C+D	2.62 × 10^–5^	1.43 × 10^–5^	0.987	0.967	6.70 × 10^–5^	4.09 × 10^–5^	0.89	0.78
	N_2_ Uptake							
A	2.44 × 10^–1^	1.50 × 10^–1^	0.461	0.18	2.51 × 10^–1^	1.56 × 10^–1^	0.288	0.01
A+B	1.35 × 10^–1^	6.42 × 10^–2^	0.926	0.749	2.11 × 10^–1^	1.17 × 10^–1^	0.671	0.34
A+B+C	3.29 × 10^–2^	1.38 × 10^–2^	0.994	0.985	1.83 × 10^–1^	8.90 × 10^–2^	0.792	0.49
A+B+C+D	8.06 × 10^–2^	2.96 × 10^–2^	0.985	0.911	1.89 × 10^–1^	9.49 × 10^–2^	0.768	0.47
A+B+C+D+E	2.99 × 10^–2^	1.84 × 10^–2^	0.991	0.988	1.04 × 10^–1^	5.62 × 10^–2^	0.936	0.84
	N_2_ Diffusion							
A	1.13 × 10^–4^	7.34 × 10^–5^	0.738	0.538	1.22 × 10^–4^	8.30 × 10^–5^	0.623	0.40
A+B	5.71 × 10^–5^	3.34 × 10^–5^	0.935	0.882	9.33 × 10^–5^	5.75 × 10^–5^	0.791	0.65
A+B+C	2.39 × 10^–5^	1.13 × 10^–5^	0.993	0.979	7.29 × 10^–5^	4.80 × 10^–5^	0.843	0.76
A+B+C+D	2.40 × 10^–5^	1.08 × 10^–5^	0.994	0.979	7.38 × 10^–5^	4.72 × 10^–5^	0.844	0.76
A+B+C+D+E	3.75 × 10^–5^	2.35 × 10^–5^	0.966	0.949	7.05 × 10^–5^	4.46 × 10^–5^	0.860	0.80

We then compared the ML-predicted adsorption and diffusion properties of He, H_2_, and CH_4_ (N_2_) with the simulation results using the 19 (20) descriptors, as listed in Table 1. Figure 3 represents the scatter plots with marginal histograms for the gas adsorption properties of MOFs. The predicting power of ML models is generally good. Figure 3a shows the highest accuracy observed for He adsorption with SRCC: 0.98 and *R*^2^: 0.99. Figure 3b also shows a quite good agreement between the ML-predicted and simulated H_2_ adsorption data of MOFs with SRCC: 0.86 and *R*^2^: 0.80 in the test set. Although the lowest *R*^2^ and SRCC values in the test set were observed for CH_4_ uptake, the predicting power of the ML model can be considered as good (*R*^2^: 0.73) in Figure 3c. In the case of CH_4_ uptake, the ML models overpredicted (underpredicted) the simulation results at low (high) uptakes of <1.5 mol/kg (>1.5 mol/kg). Figure 3d represents the high accuracy of the ML model for N_2_ uptake prediction with an *R*^2^ of 0.84 and an SRCC of 0.94 in the test set. Overall, with the lowest SRCC value of 0.86, the rankings of MOF based on the ML-predicted gas uptakes are strongly correlated with those based on the simulation results in the test set for all gases.

**Figure 4:**
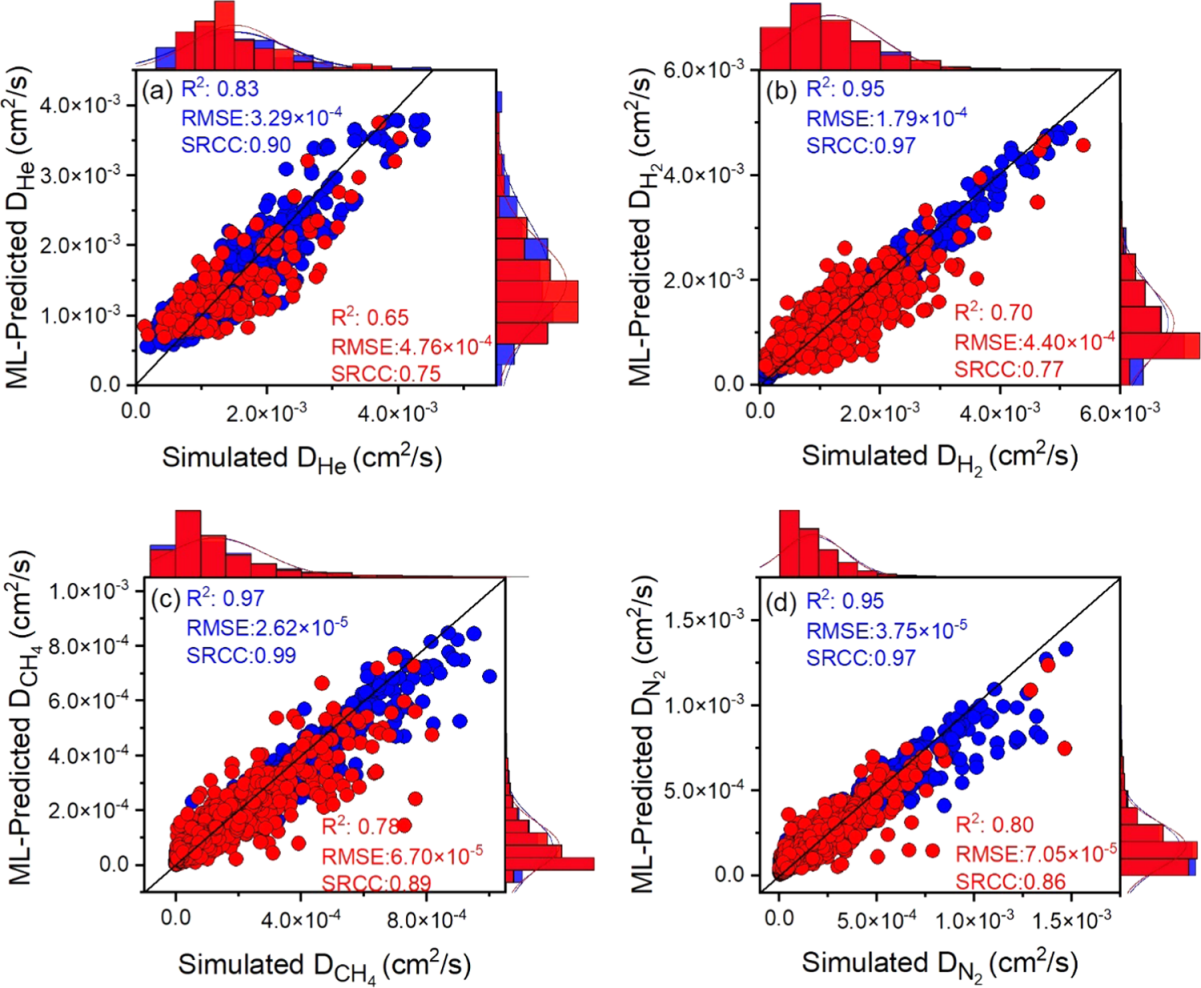
Comparison of the ML-predicted diffusion of (a) He, (b) H_2_, (c) CH_4_, and (d) N_2_ in MOFs with the simulated ones. Blue (red) symbols represent the training (test) data.

We then trained ML models to predict the gas diffusion in MOFs. *R*^2^ and SRCC values of the test set for He, H_2_, N_2_, and CH_4_ gases were computed to be in the ranges of 0.65–0.80 and 0.75–0.89, respectively, as shown in Figure 4. Some *R*^2^ and SRCC values that we collected from the literature are as follows: *R*^2^ values for the three ML models developed for predicting N_2_ diffusivity (O_2_/N_2_ adsorption selectivity) in MOFs were reported to be in the range of 0.74–0.80 (0.32–0.55).^61^*R*^2^ (SRCC) values of ML models trained for predicting the C_3_H_8_ uptake, Henry’s constant of C_3_H_8_, and adsorption selectivity for C_3_H_8_/C_3_H_6_ separation were reported as 0.82 (0.89), 0.93 (0.96), and 0.73 (0.76) in the test set, respectively.^24^ As discussed before, gas diffusivity depends on more complex parameters compared to gas uptakes; thus, ML models predicting diffusivity in MOFs have not been widely studied. In our recent work, *R*^2^ of ML models were reported as 0.74 for N_2_ diffusivity in MOFs and 0.76 for O_2_ diffusivity in MOFs for O_2_/N_2_ separation.^30^ Overall, we showed that although the level of agreement between the ML predictions and simulation results is lower for the gas diffusivities compared to that for the gas uptakes, the accuracy of ML models is still acceptable based on the previous literature. The predicting power of ML models for He and H_2_ diffusivities shown in Figure 4a,b is lower than that for N_2_ and CH_4_ diffusivities, as shown in Figure 4c,d. Among the diffusivities of He, H_2_, N_2_, and CH_4_ gases, the best prediction was made for N_2_ diffusivities (Figure 4d), resulting in a high *R*^2^ of 0.80, an SRCC of 0.86, and a low RMSE of 7.1 × 10^–5^. This can be attributed to the fact that gas molecules with smaller kinetic diameters (He, H_2_) diffuse easily, with less dependency on the pore geometry of the MOF, compared to molecules with larger kinetic diameters (N_2_, CH_4_).

**Figure 5:**
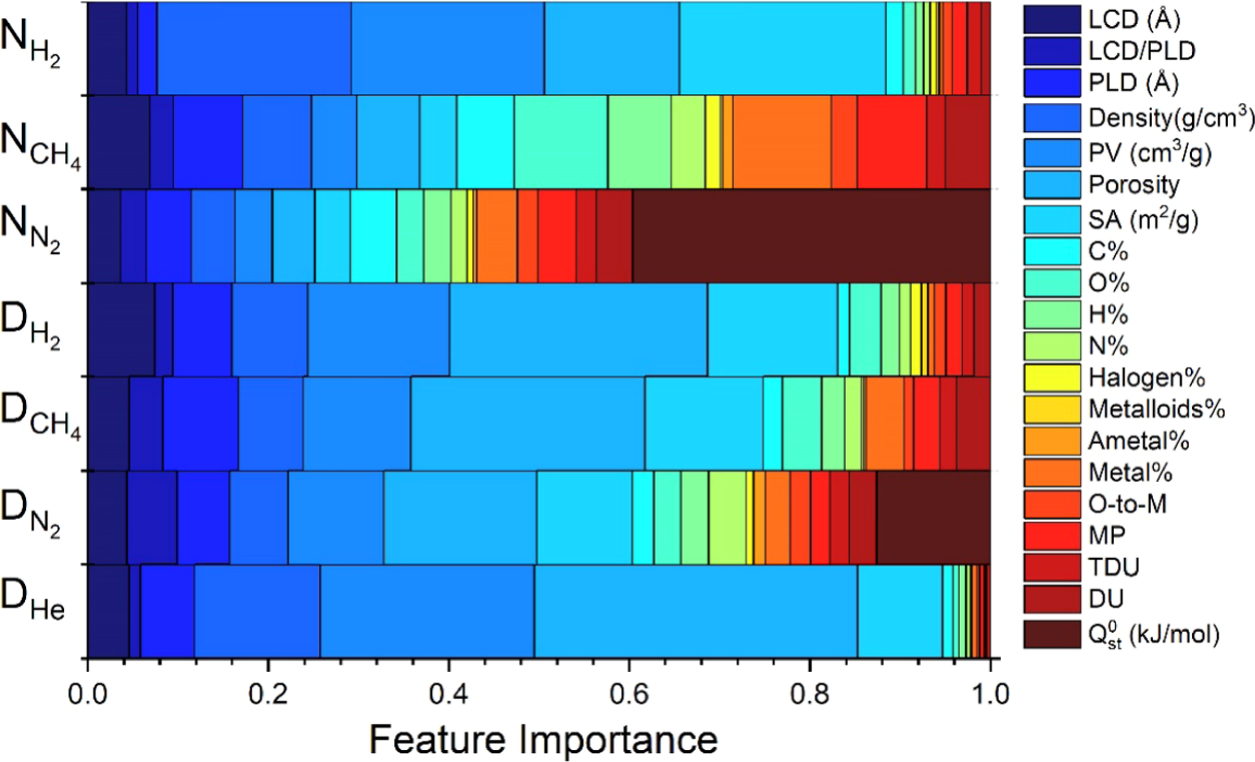
Feature importance for the gas adsorption and diffusion properties of MOFs. The width range of each color shows the importance of the related feature. The colors were taken from the same palette for each group.

Figure 5 shows the feature importance analysis for all target variables. The relative importance of the features varies across the ML models developed to predict the adsorption and diffusion properties of gases in MOFs. While the pore size and geometry are more important for training ML models for H_2_ adsorption, atom types and chemical descriptors significantly affect CH_4_ and N_2_ adsorption. For the development of the ML model to predict N_2_ uptake, *Q*_st_^0^ was also considered as the energy descriptor and played the most important role in describing the N_2_ uptake. The importance of the pore size and geometry in the models predicting gas diffusivities is generally higher compared to those predicting gas uptakes. Especially, the importance of the pore size ratio (LCD/PLD) used in the ML models to predict N_2_ and CH_4_ diffusivities is generally more pronounced than those used to estimate the gas uptakes. Porosity is the most important descriptor to accurately predict N_2_ diffusivities, and *Q*_st_^0^ also has an impact. Overall, we concluded that physical features such as pore size and geometry of MOFs are important to train the ML models for both gas adsorption and diffusion data. Compared to the gas diffusivity, predictions for gas uptakes are much more affected by the inclusion of chemical descriptors, atom types, and energy descriptors in the ML models. We finally note that He uptake was not shown in Figure 5 because ML models for all target data except He uptake were trained with tree-based algorithms, which were constructed using the Gini index that determines the relative importance of features.

**Figure 6:**
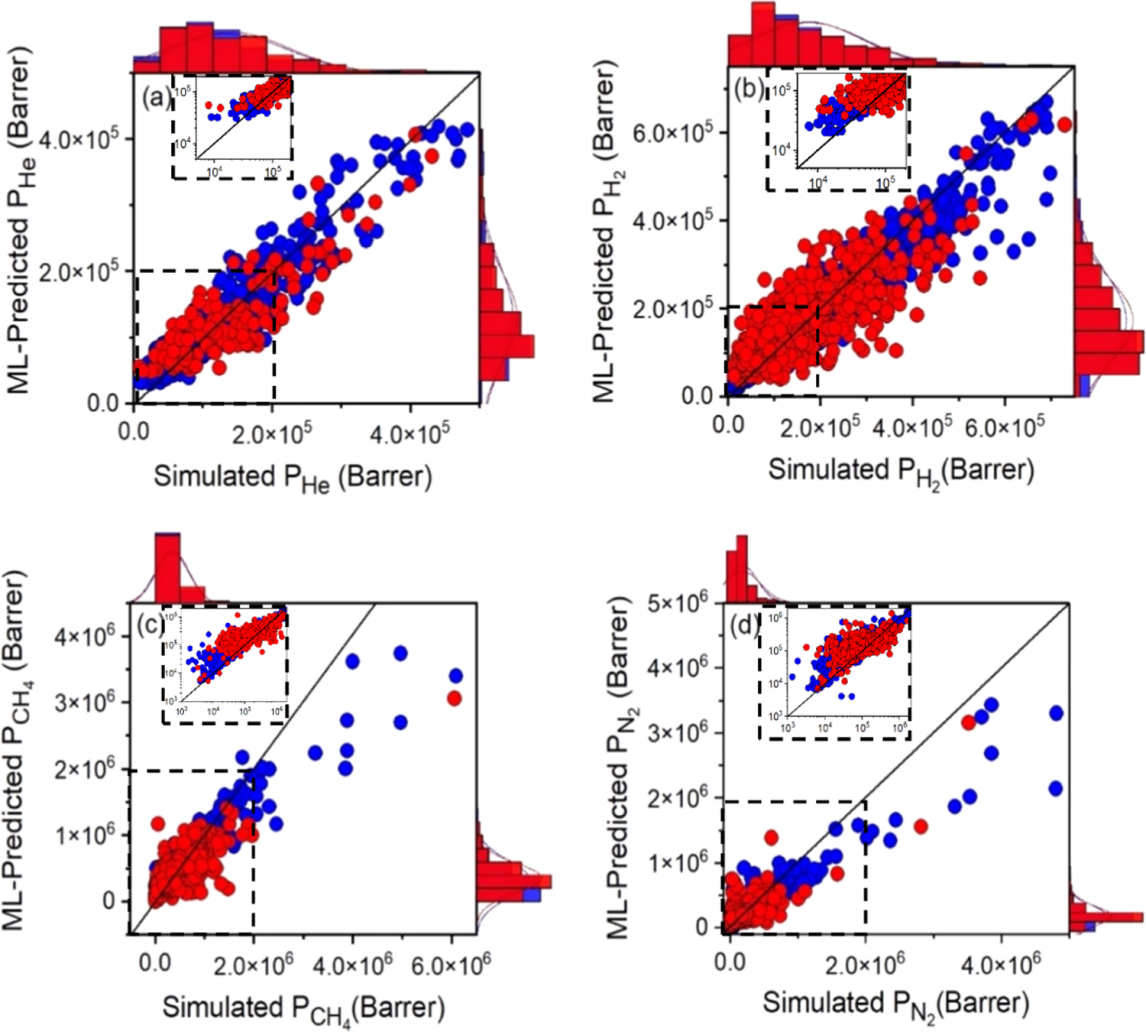
Comparison of the ML-predicted (a) He, (b) H_2_, (c) CH_4_, and (d) N_2_ permeability of the MOFs with the simulated ones. Blue (red) symbols represent the training (test) data. The inset figures represent the data in the dashed boxes in the log–log scale.

Next, we calculated the ML-predicted gas permeabilities and compared them with the simulated permeabilities in Figure 6. We note that the term “ML-predicted permeability” was used for the permeability that was calculated using ML-predicted adsorption and diffusion data and “simulated permeability” was used for the permeability that was calculated using simulated gas adsorption and diffusion data. To the best of our knowledge, these are the first ML models developed to predict He, H_2_, N_2_, and CH_4_ permeabilities of MOFs at realistic conditions, 1 bar, 298 K. Figure 6a,b shows that there is a good agreement between ML-predicted and simulated permeabilities, especially for He and H_2_. Figure 6c,d presents that ML-predicted N_2_ and CH_4_ permeabilities are generally lower than simulated ones in the high gas permeability range (>10^6^ Barrer), but the agreement is good in the low permeability range. We also showed the ratios of the ML-predicted gas uptakes, diffusivities, and permeabilities to the simulated ones for the training and test sets in Figure S8. The average ratio is close to unity for gas uptakes, indicating the good agreement between ML and simulations. The range of the ratios (0.11–47.5) is larger for gas diffusivities; therefore, deviations between ML-predicted and simulated gas permeabilities were more observable compared to those between uptakes and diffusivities.

In addition to the gas permeability, selectivity is an important metric to assess membranes’ separation performances. We calculated He/H_2_, He/N_2_, He/CH_4_, H_2_/N_2_, H_2_/CH_4_, and N_2_/CH_4_ membrane selectivities of MOFs. Since two different gas permeability data are needed to calculate the membrane selectivity of an MOF, we calculated selectivities only for the MOFs commonly existing in the test sets of both gases. Figure S9 shows that there is good agreement between the ML-predicted and simulated membrane selectivities of MOFs for six different gas separations that we considered. Overall, the results so far suggest that ML models that we developed in this work for predicting gas adsorption and diffusion properties of MOFs can accurately estimate gas permeabilities and selectivities of MOF membranes and therefore they would be very useful for the initial assessment of MOF membranes for a target gas separation before the experimental efforts.

### 3.3 Predictions of ML Models for MOF/Polymer MMMs

**Figure 7:**
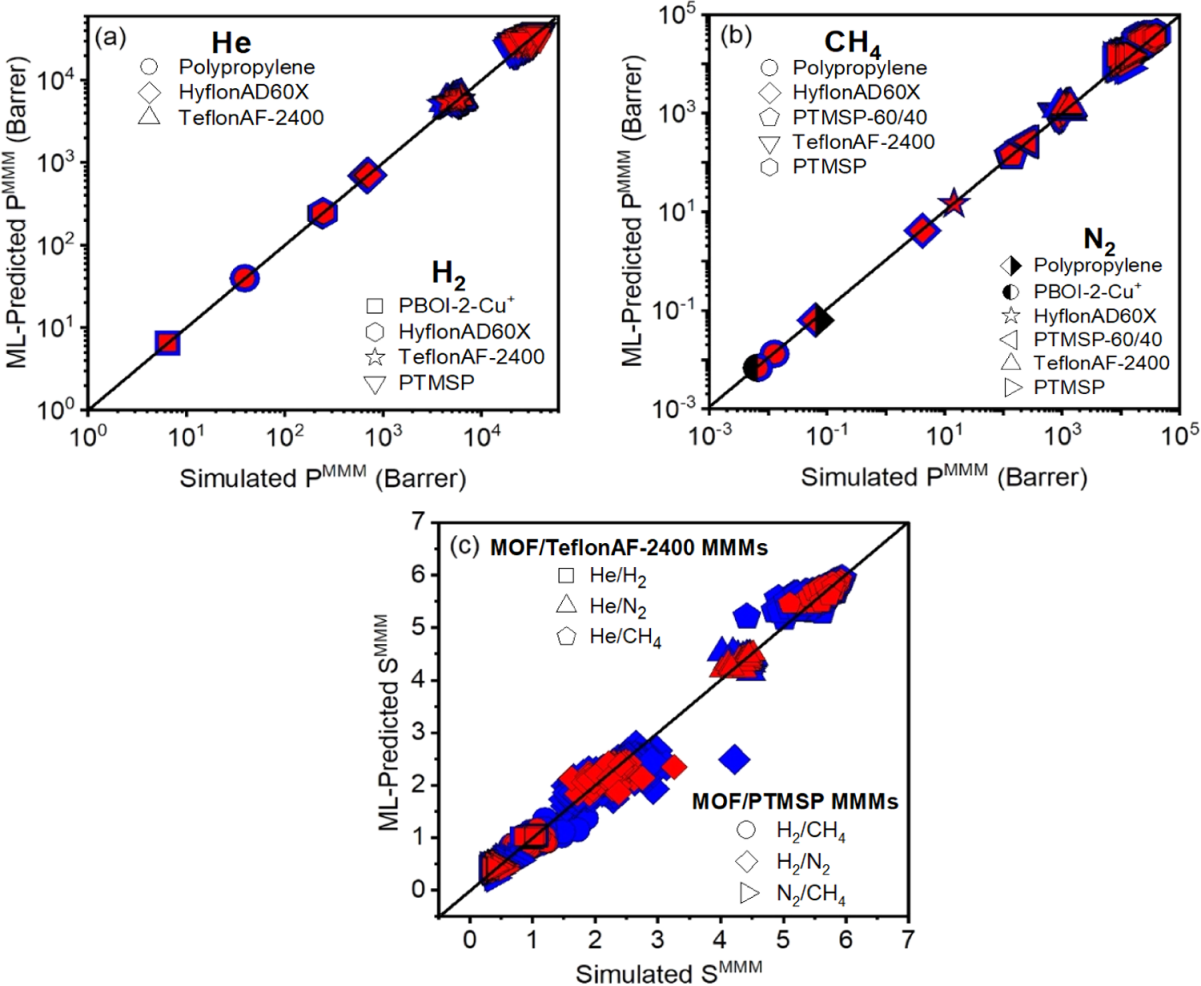
Comparison of the ML-predicted and simulated (a) He and H_2_ and (b) N_2_ and CH_4_ permeabilities of MOF-based MMMs. (c) Comparison of the ML-predicted and simulated selectivities of MOF/polymer MMMs for He/H_2_, He/N_2_, He/CH_4_, H_2_/CH_4_, H_2_/N_2_, and N_2_/CH_4_ separations. Blue (red) symbols represent the training (test) set. The data for the test set are shown with smaller symbols than those for the training set in panels (a–c) to make all data visible.

Motivated by the good agreement between the ML-predicted and simulated gas permeabilities of pristine MOFs, we calculated the permeability and selectivity of MOF/polymer MMMs using both the ML models and results of molecular simulations. Figure 7 shows that there is good agreement between the ML-predicted and simulated gas permeabilities and selectivities of MMMs. ML predictions were found to be in strong agreement with the simulations for the MMMs composed of polymers having low or medium gas permeability (polypropylene, PBOI-2-Cu^+^). On the other hand, the accuracy of ML predictions was found to be lower for the MMMs composed of highly permeable polymers (TeflonAF-2400, PTMSP). Figure 7a shows that ML-predicted permeabilities of MMMs are in a wider range when the polymers having high gas permeabilities (>10^3^ Barrer) are used compared to those having polymers with relatively low permeabilities (<10^3^ Barrer). The most significant difference between the ML-predicted and simulated permeabilities was observed for MMMs composed of two highly permeable polymers, TeflonAF-2400 and PTMSP. Thus, we focused on MOF/TeflonAF-2400 and MOF/PTMSP MMMs in Figure 7c.

For He-related separations (He/H_2_, He/N_2_, and He/CH_4_), the ML-predicted and simulated selectivities of MMMs are in strong agreement. For example, the ratios of the ML-predicted He/CH_4_ selectivity over the simulated one for MOF/TeflonAF-2400 MMMs in the test set were 0.98–1.07, suggesting that our ML models can accurately predict the He/CH_4_ selectivity of these MMMs. The ratios of the ML-predicted N_2_/CH_4_, H_2_/CH_4_, and H_2_/N_2_ selectivities over the simulated selectivity in the test set were calculated to be in a wider range, 0.70–1.33, 0.72–1.29, and 0.72–1.31, respectively, for MOF/PTMSP MMMs. The ML-predicted selectivity of MMMs for most MOFs in the test set was generally lower than the simulated selectivity when the polymer having a high gas permeability was used. This is expected due to the overestimation of the gas permeabilities by the ML models, as discussed in Figure 7a,b. We note that we considered the common MOFs in training and test sets for each gas pair; therefore, the number of MOFs used for selectivity predictions is lower than those used for permeability predictions. For example, 677 and 2715 MOFs were used to develop ML models for predicting He and H_2_ permeabilities but a much smaller number of MOFs, 382 and 28 MOFs (in the training and test sets, respectively), was used for the evaluation of the ML models to predict the He/H_2_ selectivity of the MMMs.

### 3.4 Comparing ML Predictions with Experimental Data

**Figure 8:**
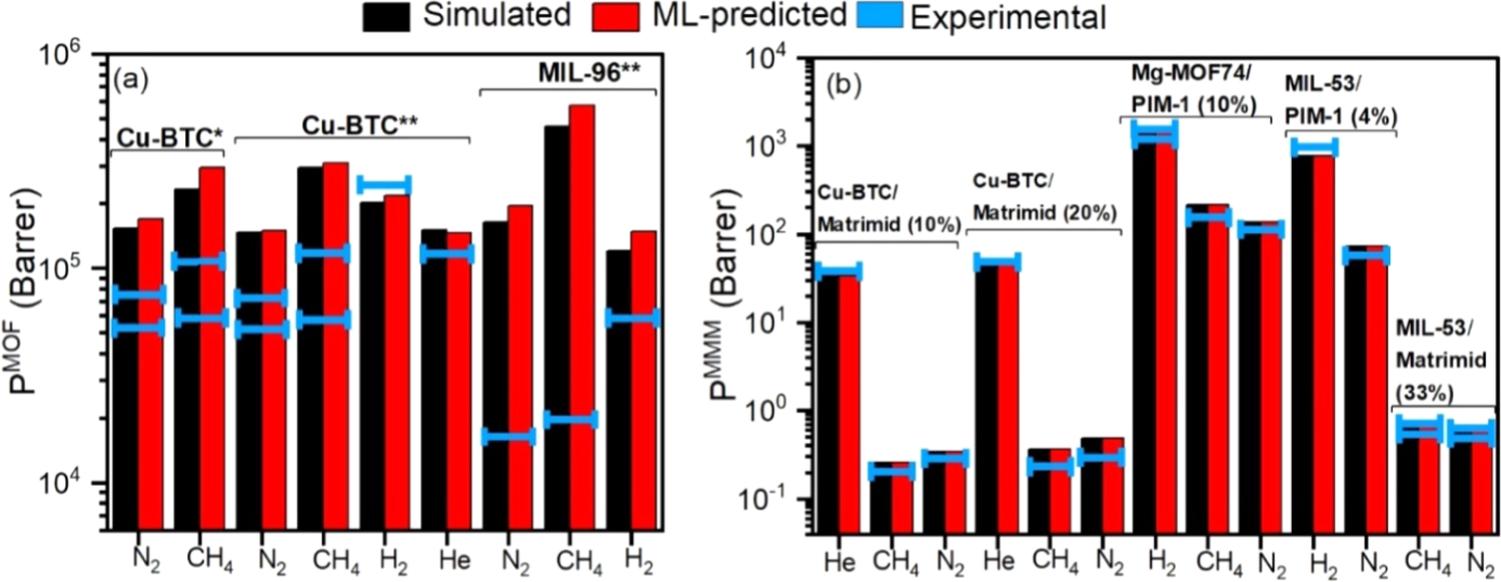
Comparison of ML-predicted and simulated gas permeabilities with the available experimental data for (a) MOF membranes and (b) MOF/polymer MMMs. Blue lines show the experimental gas permeabilities collected from the literature. The number of the blue lines on each column represents the number of experimental data at (a) 1 bar, 298 K, for MOF membranes and (b) 0.5–5 bar, 298–308 K, for MOF/polymer MMMs. The values in parentheses in panel (b) represent the volume fraction of MOF fillers. * (**) represents that the MOF was taken from the test (training) set.

We so far compared the ML-predicted and simulated gas separation performances of MOF membranes and MOF/polymer MMMs. Despite the scarcity in the reported experimental gas permeabilities of the pure MOF membranes, there are several MOF/polymer MMMs that were tested for different gas separations in the literature.^12^ To make a comprehensive comparison between ML predictions, molecular simulations, and experiments, we collected the experimental He, H_2_, N_2_, and CH_4_ permeabilities of the MOF membranes and MOF-based MMMs from the literature. We note that simulated and ML-predicted gas permeabilities of MOF-based MMMs were calculated using the same filler loading as the corresponding experiments. These experimental permeability data of MOF membranes and MMMs are presented in Figure 8 together with our corresponding ML predictions and simulation results. Figure 8a represents the ML-predicted, simulated, and experimentally measured gas permeabilities of two MOFs, Cu-BTC and MIL-96, which were in our material database used for training ML models. Simulated and ML-predicted gas permeabilities of the MOFs strongly agree, but they generally overestimate experimental gas permeabilities of Cu-BTC^62,63^ and MIL-96.^64^ As previously discussed in the literature,^16^ MOFs were modeled as perfect, defect-free crystal structures in the molecular simulations, which leads to high permeabilities, but defects may exist in the fabricated membranes.

Even though our ML models somehow overpredicted the gas permeabilities of MOF membranes, the rankings of MOFs based on the ML-predicted gas adsorption and diffusion data agree well with the simulated ones (SRCC in the range of 0.75–0.98), as discussed above. These rankings can be useful to the experimentalists for selecting the best candidates from a large group of MOFs for membrane fabricating and testing. Figure 8b shows He, H_2_, N_2_, and CH_4_ permeabilities of three different MMMs^65−67^ composed of well-known MOFs (Cu-BTC, Mg-MOF74, and MIL-53) with different volume fractions and polymers (Matrimid, PIM-1). Simulated, ML-predicted, and experimental gas permeabilities all agree well, showing the strength of our ML models to predict the gas separation performances of the MOF/polymer MMMs. This is an important result because considering the existence of thousands of MOFs and hundreds of polymers, a theoretically infinite number of MOF/polymer MMMs can be generated and accurate estimates for the gas separation performances of all of these possible MMMs using the ML models that we develop will significantly accelerate the design and fabrication of new MMMs for a variety of gas separations.

### 3.5 Transferability of ML Models

**Figure 9:**
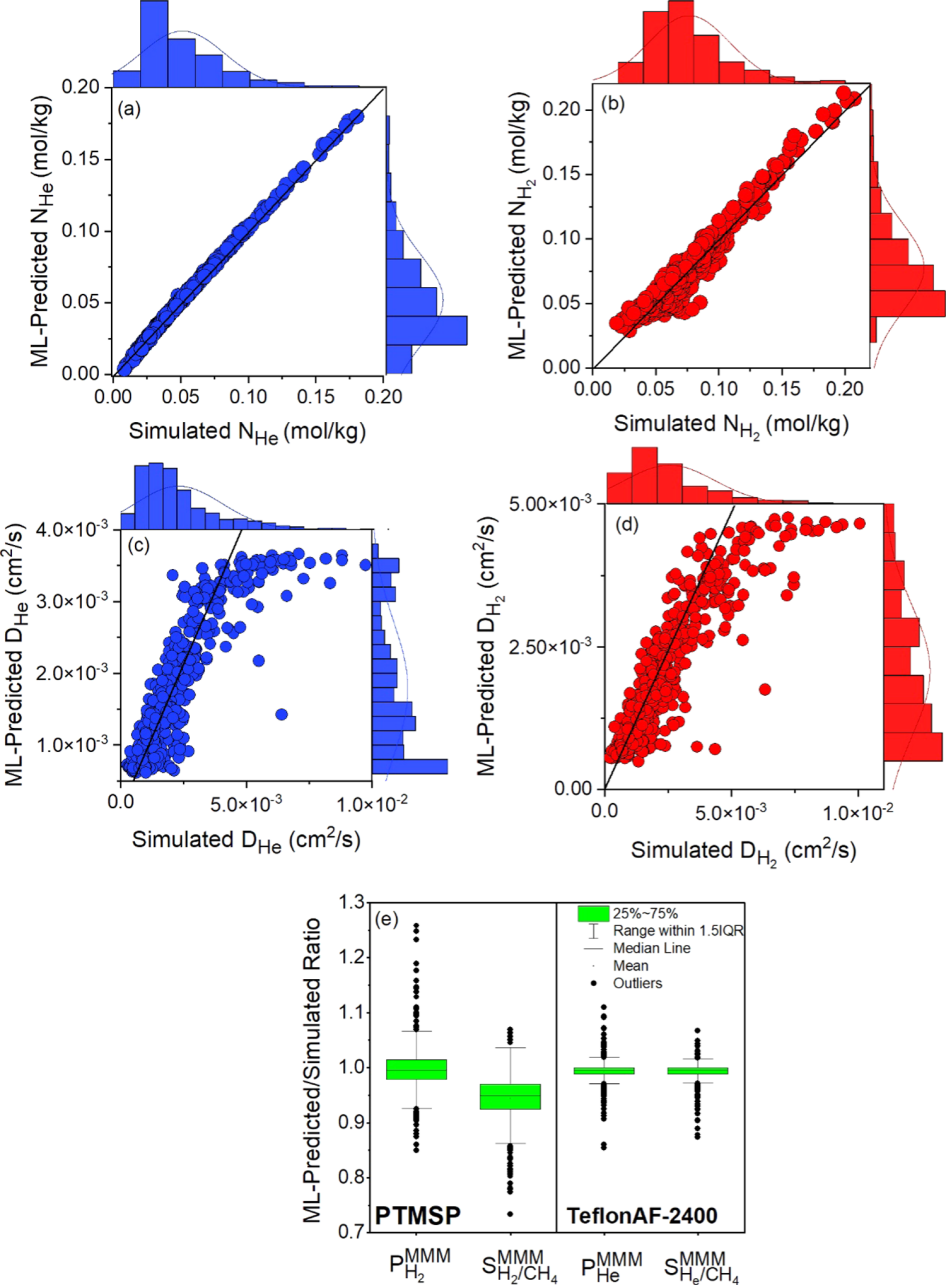
Comparison of ML-predicted (a, c) uptake and (b, d) diffusivity for He and H_2_ in 500 hMOFs with the simulated ones. The black line represents *x* = *y*. (e) The ratio of ML-predicted permeability and selectivity values to that of simulated ones for 1000 MMMs. The left (right) side of the figure represents the results related to hMOF/PTMSP (hMOF/TeflonAF-2400) MMMs. Boxes show the quartiles of the data set, while whiskers extend to show the rest of the distribution, except for outliers that were defined as values more than 1.5IQR (IQR = interquartile range) from either end of the box.

One of the main advantages of developing ML models for a set of materials is the ability to transfer these models to a different set of new, unexplored materials and make accurate predictions for these unseen materials. Motivated by the good agreement between the ML, molecular simulations, and experiments, we used our ML models, which were originally developed for experimentally synthesized MOFs, to predict the separation performances of hMOFs. hMOFs have not been synthesized yet; thus, no experimental gas adsorption, diffusion, and/or permeability data is available for them. After determining the ML-predicted adsorption and diffusion properties of hMOFs for He, H_2_, N_2_, and CH_4_, we performed GCMC and MD simulations for hMOFs to compare ML predictions with simulation results. The heatmap with the Pearson correlations across different features of hMOFs is shown in Figure S10, which indicates that the correlations are generally like those observed for MOFs. Figure 9 shows the comparison of the ML-predicted and simulated uptakes and diffusivities of He and H_2_ in 500 hMOFs. We also computed the MAE, *R*^2^, SRCC, and RMSE of the ML-predicted gas uptake and diffusivity in hMOFs, as shown in Table S5. Figure 9a,b shows that the ML-predicted He and H_2_ uptakes agree well with the corresponding simulated uptakes. On the other hand, ML-predicted uptakes of most hMOFs for CH_4_ and N_2_ (71 and 88% of all hMOFs, respectively) are higher than the simulated uptakes, as shown in Figure S11a,b. It is important to note that the ranges of simulated He, H_2_, and CH_4_ uptakes of hMOFs are similar to those predicted by ML models in MOFs (as shown in Figure 3), but the range of simulated N_2_ uptakes in hMOFs is narrower than that in MOFs.

In Figure 9c,d, it is shown that for most of the hMOFs, the ML-predicted He and H_2_ diffusivities are similar to the simulated ones. The ML models consistently underestimated the simulated gas diffusivities in a small number of hMOFs exhibiting diffusivities above certain values (>4 × 10^–3^ cm^2^/s for He diffusivities and >5 × 10^–3^ cm^2^/s for H_2_ diffusivities). This can be attributed to the fact that the tree-based algorithm, by construction, suffers from the extrapolation of unseen data. In other words, they cannot reach the trends for cases lying outside the training data.^68^ Similar results were observed for the self-diffusivity predictions of N_2_ and CH_4_, as shown in Figure S11c,d. Overall, these results showed that ML models that we trained for MOFs can predict the gas uptake and diffusivities of hMOFs fairly well, suggesting the transferability of ML models to different membrane materials.

We finally investigated the applicability of ML models to predict the gas permeability and selectivity of hMOF/polymer MMMs. Since the lowest predictability power of ML models were obtained for the MOF/polymer MMMs having highly permeable polymers (previously shown in Figure 7a,b), we focused on 1000 hMOF/polymer MMMs composed of 500 different hMOFs and 2 highly permeable polymers, TeflonAF-2400 and PTMSP, for He/CH_4_ and H_2_/CH_4_ separations. Figure 9e shows the ratio of the ML-predicted H_2_ (He) permeability and H_2_/CH_4_ (He/CH_4_) selectivity of 500 hMOF/PTMSP (hMOF/TeflonAF-2400) MMMs to the simulated ones. For He/CH_4_ separation, the ranges of these ratios for hMOF/Teflon MMMs were found to be between 0.85 and 1.1 for He permeability and 0.87 and 1.07 for He/CH_4_ selectivity. Similarly, even if we studied one of the most permeable polymers (PTMSP), the ratios were found to be close to unity for H_2_ permeability (0.85–1.26) and H_2_/CH_4_ selectivity (0.73–1.07). Thus, we can conclude that the ML models developed to predict the gas uptake and diffusivity of MOFs lead to accurate gas permeability and selectivity predictions for the unseen hMOF-based MMMs.

## 4 Conclusions

In this study, we investigated the gas separation performances of MOF membranes and MOF/polymer MMMs by combining molecular simulations and machine learning for six different separations, He/H_2_, He/N_2_, He/CH_4_, H_2_/N_2_, H_2_/CH_4_, and N_2_/CH_4_. Using 20 different physical and chemical and energy-based descriptors of MOFs, we developed eight different ML models including LassoLarsCV, ETR, GBR, and RFR algorithms to predict the uptake and diffusivity of He, H_2_, N_2_, and CH_4_ in MOFs. The accuracy of ML models was found to be high for both the gas uptake and diffusion properties of MOFs leading to an *R*^2^ of 0.73–0.99 and 0.65–0.80, respectively, and an SRCC of 0.86–0.98 and 0.75–0.89, respectively. The feature importance analysis revealed that the physical properties such as porosity are more critical for the accurate prediction of gas adsorption and diffusion data of MOFs compared to the chemical descriptors such as atom types and degree of unsaturation. ML-predicted gas uptake and diffusivity data were used to compute He, H_2_, CH_4_, and N_2_ permeabilities of a total of 5249 MOF membranes and a total of 31,494 MOF/polymer MMMs, and the results were shown to be in good agreement with the permeabilities computed from the simulations. Comparisons between the ML-predicted, simulated, and experimentally reported gas permeabilities of different MOF membranes and MOF/polymer MMMs showed that our ML models will be very useful to estimate gas separation performances of MOF-based membranes in a rapid and accurate manner. Finally, the transferability of ML models developed for real MOFs to hMOFs was examined and results showed that ML models can successfully predict gas permeabilities of hMOFs/polymer MMMs. Overall, the ML models that we developed in this work to predict the gas uptake and diffusion properties of MOFs will be very useful to evaluate the gas separation performance of a large number and variety of MOF membranes and MOF/polymer MMMs by saving an enormous amount of computational time for molecular simulations and huge amounts of efforts for the experimental fabrication and testing of membranes. These rapid and accurate models will also be beneficial for allocating experimental efforts, resources, and time to the most promising membrane materials.

## Supporting Information Available

The cutoff threshold values; the potential parameters of gas models; gas permeability of polymers; distribution of features of hMOFs; distribution of features based on training and test sets for He, H_2_, CH_4_, and N_2_; the effects of MOF features on their H_2_ and N_2_ adsorption and He, CH_4_, H_2_, and N_2_ diffusion; correlation heatmap for the descriptors of MOFs; ML models and parameters based on the gas adsorption and diffusion properties; the ratio of the ML-predicted uptake; diffusivity and permeability to the simulated ones for He, H_2_, N_2_, and CH_4_ gases; comparison of ML-predicted and simulated selectivities of MOFs in the test set for He/H_2_, He/N_2_, He/CH_4_, H_2_/N_2_, H_2_/CH_4_, and N_2_/CH_4_ separations; correlation heatmap for the descriptors of hMOFs; MAE, RMSE, SRCC, and *R*^2^ of ML-predicted gas uptake and diffusivity in hMOFs; comparison of ML-predicted and simulated uptakes and diffusivities for CH_4_ and N_2_ gases of hMOFs; and ML scripts available at https://github.com/hdaglar/MOF-basedMMMs_ML (PDF)

## References

[ref1] Farha O. K.; Eryazici I.; Jeong N. C.; Hauser B. G.; Wilmer C. E.; Sarjeant A. A.; Snurr R. Q.; Nguyen S. T.; Yazaydın A.Ö.; Hupp J. T. Metal–Organic Framework Materials with Ultrahigh Surface Areas: Is the Sky the Limit?. J. Am. Chem. Soc. 2012, 134, 15016–15021. 10.1021/ja3055639. 22906112

[ref2] Gomollón-Bel F. Ten Chemical Innovations That Will Change Our World: IUPAC Identifies Emerging Technologies in Chemistry with Potential to Make Our Planet More Sustainable. Chem. Int. 2019, 41, 12–17. 10.1515/ci-2019-0203.

[ref3] The Cambridge Structural Database (CSD), UK. Available from: https://www.ccdc.cam.ac.uk/CCDCStats/Stats.

[ref4] Keskin S.; van Heest T. M.; Sholl D. S. Can Metal–Organic Framework Materials Play a Useful Role in Large-scale Carbon Dioxide Separations?. ChemSusChem 2010, 3, 879–891. 10.1002/cssc.201000114. 20730980

[ref5] Han X.; Godfrey H. G. W.; Briggs L.; Davies A. J.; Cheng Y.; Daemen L. L.; Sheveleva A. M.; Tuna F.; McInnes E. J. L.; Sun J.; Drathen C.; George M. W.; Ramirez-Cuesta A. J.; Thomas K. M.; Yang S.; Schröder M. Reversible Adsorption of Nitrogen Dioxide within a Robust Porous Metal-Organic Framework. Nat. Mater. 2018, 17, 691–696. 10.1038/s41563-018-0104-7. 29891889

[ref6] Chen Z.; Mian M. R.; Lee S.-J.; Chen H.; Zhang X.; Kirlikovali K. O.; Shulda S.; Melix P.; Rosen A. S.; Parilla P. A.; Gennett T.; Snurr R. Q.; Islamoglu T.; Yildirim T.; Farha O. K. Fine-Tuning a Robust Metal–Organic Framework Toward Enhanced Clean Energy Gas Storage. J. Am. Chem. Soc. 2021, 143, 18838–18843. 10.1021/jacs.1c08749. 34752071

[ref7] Bucior B. J.; Bobbitt N. S.; Islamoglu T.; Goswami S.; Gopalan A.; Yildirim T.; Farha O. K.; Bagheri N.; Snurr R. Q. Energy-based Descriptors to Rapidly Predict Hydrogen Storage in Metal–Organic Frameworks. Mol. Syst. Des. Eng. 2019, 4, 162–174. 10.1039/C8ME00050F.

[ref8] Ding Y. Perspective on Gas Separation Membrane Materials from Process Economics Point of View. Ind. Eng. Chem. Res. 2020, 59, 556–568. 10.1021/acs.iecr.9b05975.

[ref9] Shekhah O.; Chernikova V.; Belmabkhout Y.; Eddaoudi M. Metal–Organic Framework Membranes: From Fabrication to Gas Separation. Crystals 2018, 8, 412 10.3390/cryst8110412.

[ref10] Daglar H.; Keskin S. Recent Advances, Opportunities, and Challenges in High-throughput Computational Screening of MOFs for Gas Separations. Coord. Chem. Rev. 2020, 422, 213470 10.1016/j.ccr.2020.213470.

[ref11] Demir H.; Aksu G. O.; Gulbalkan H. C.; Keskin S. MOF Membranes for CO_2_ Capture: Past, Present and Future. Carbon Capture Sci. Technol. 2022, 2, 100026 10.1016/j.ccst.2021.100026.

[ref12] Daglar H.; Erucar I.; Keskin S. Recent Advances in Simulating Gas Permeation through MOF Membranes. Mater. Adv. 2021, 2, 5300–5317. 10.1039/D1MA00026H. 34458845 PMC8366394

[ref13] Lin W.-q.; Xiong X.-l.; Liang H.; Chen G.-h. Multiscale Computational Screening of Metal–Organic Frameworks for Kr/Xe Adsorption Separation: A Structure–Property Relationship-Based Screening Strategy. ACS Appl. Mater. Interfaces 2021, 13, 17998–18009. 10.1021/acsami.1c02257. 33821608

[ref14] Daglar H.; Erucar I.; Keskin S. Exploring the Performance Limits of MOF/polymer MMMs for O_2_/N_2_ Separation Using Computational Screening. J. Membr. Sci. 2021, 618, 118555 10.1016/j.memsci.2020.118555.

[ref15] Avci G.; Erucar I.; Keskin S. Do New MOFs Perform Better for CO_2_ Capture and H_2_ Purification? Computational Screening of the Updated MOF Database. ACS Appl. Mater. Interfaces 2020, 12, 41567–41579. 10.1021/acsami.0c12330. 32818375 PMC7591111

[ref16] Daglar H.; Keskin S. Computational Screening of Metal–Organic Frameworks for Membrane-based CO_2_/N_2_/H_2_O Separations: Best Materials for Flue Gas Separation. J. Phys. Chem. C 2018, 122, 17347–17357. 10.1021/acs.jpcc.8b05416. PMC6077770 30093931

[ref17] Altintas C.; Keskin S. Molecular Simulations of MOF Membranes and Performance Predictions of MOF/Polymer Mixed Matrix Membranes for CO_2_/CH_4_ Separations. ACS Sustainable Chem. Eng. 2019, 7, 2739–2750. 10.1021/acssuschemeng.8b05832. 30701144 PMC6344032

[ref18] Altintas C.; Altundal O. F.; Keskin S.; Yildirim R. Machine Learning Meets with Metal Organic Frameworks for Gas Storage and Separation. J. Chem. Inf. Model. 2021, 61, 2131–2146. 10.1021/acs.jcim.1c00191. 33914526 PMC8154255

[ref19] Borboudakis G.; Stergiannakos T.; Frysali M.; Klontzas E.; Tsamardinos I.; Froudakis G. E. Chemically Intuited, Large-scale Screening of MOFs by Machine Learning Techniques. npj Comput. Mater. 2017, 3, 40 10.1038/s41524-017-0045-8.

[ref20] Dureckova H.; Krykunov M.; Aghaji M. Z.; Woo T. K. Robust Machine Learning Models for Predicting High CO_2_ Working Capacity and CO_2_/H_2_ Selectivity of Gas Adsorption in Metal Organic Frameworks for Precombustion Carbon Capture. J. Phys. Chem. C 2019, 123, 4133–4139. 10.1021/acs.jpcc.8b10644.

[ref21] Fernandez M.; Boyd P. G.; Daff T. D.; Aghaji M. Z.; Woo T. K. Rapid and Accurate Machine Learning Recognition of High Performing Metal Organic Frameworks for CO_2_ Capture. J. Phys. Chem. Lett. 2014, 5, 3056–3060. 10.1021/jz501331m. 26278259

[ref22] Li L.; Shi Z.; Liang H.; Liu J.; Qiao Z. Machine Learning-Assisted Computational Screening of Metal-Organic Frameworks for Atmospheric Water Harvesting. Nanomaterials 2022, 12, 159 10.3390/nano12010159. 35010109 PMC8746952

[ref23] Cho E. H.; Deng X.; Zou C.; Lin L.-C. Machine Learning-Aided Computational Study of Metal–Organic Frameworks for Sour Gas Sweetening. J. Phys. Chem. C 2020, 124, 27580–27591. 10.1021/acs.jpcc.0c09073.

[ref24] Tang H.; Xu Q.; Wang M.; Jiang J. Rapid Screening of Metal–Organic Frameworks for Propane/Propylene Separation by Synergizing Molecular Simulation and Machine Learning. ACS Appl. Mater. Interfaces 2021, 13, 53454–53467. 10.1021/acsami.1c13786. 34665615

[ref25] Li Z.; Bucior B. J.; Chen H.; Haranczyk M.; Siepmann J. I.; Snurr R. Q. Machine Learning Using Host/guest Energy Histograms to Predict Adsorption in Metal–organic Frameworks: Application to Short Alkanes and Xe/Kr Mixtures. J. Chem. Phys. 2021, 155, 014701 10.1063/5.0050823. 34241399

[ref26] Zhou M.; Vassallo A.; Wu J. Toward the Inverse Design of MOF Membranes for Efficient D_2_/H_2_ Separation by Combination of Physics-based and Data-Driven Modeling. J. Membr. Sci. 2020, 598, 117675 10.1016/j.memsci.2019.117675.

[ref27] Yang W.; Liang H.; Peng F.; Liu Z.; Liu J.; Qiao Z. Computational Screening of Metal–Organic Framework Membranes for the Separation of 15 Gas Mixtures. Nanomaterials 2019, 9, 467 10.3390/nano9030467. PMC6474094 30897779

[ref28] Cao X.; He Y.; Zhang Z.; Sun Y.; Han Q.; Guo Y.; Zhong C. Predicting of Covalent Organic Frameworks for Membrane-based Isobutene/1, 3-Butadiene Separation: Combining Molecular Simulation and Machine Learning. Chem. Res. Chin. Univ. 2022, 38, 421–427. 10.1007/s40242-022-1452-z.

[ref29] Bai X.; Shi Z.; Xia H.; Li S.; Liu Z.; Liang H.; Liu Z.; Wang B.; Qiao Z. Machine-Learning-Assisted High-Throughput computational screening of Metal–Organic framework membranes for hydrogen separation. Chem. Eng. J. 2022, 446, 136783 10.1016/j.cej.2022.136783.

[ref30] Orhan I. B.; Daglar H.; Keskin S.; Le T. C.; Babarao R. Prediction of O_2_/N_2_ Selectivity in Metal–Organic Frameworks via High-Throughput Computational Screening and Machine Learning. ACS Appl. Mater. Interfaces 2022, 14, 736–749. 10.1021/acsami.1c18521. 34928569

[ref31] Qian Q.; Asinger P. A.; Lee M. J.; Han G.; Rodriguez K. M.; Lin S.; Benedetti F. M.; Wu A. X.; Chi W. S.; Smith Z. P. MOF-based Membranes for Gas Separations. Chem. Rev. 2020, 120, 8161–8266. 10.1021/acs.chemrev.0c00119. 32608973

[ref32] Budhathoki S.; Ajayi O.; Steckel J. A.; Wilmer C. E. High-throughput computational prediction of the cost of carbon capture using mixed matrix membranes. Energy Environ. Sci. 2019, 12, 1255–1264. 10.1039/C8EE02582G.

[ref33] Daglar H.; Aydin S.; Keskin S. MOF-based MMMs Breaking the Upper Bounds of Polymers for a Large Variety of Gas Separations. Sep. Purif. Technol. 2022, 281, 119811 10.1016/j.seppur.2021.119811.

[ref34] Yan T.; Lan Y.; Tong M.; Zhong C. Screening and Design of Covalent Organic Framework Membranes for CO_2_/CH_4_ Separation. ACS Sustainable Chem. Eng. 2018, 7, 1220–1227.

[ref35] Azar A. N. V.; Velioglu S.; Keskin S. Large-scale Computational Screening of Metal Organic Framework (MOF) Membranes and MOF-based Polymer Membranes for H_2_/N_2_ Separations. ACS Sustainable Chem. Eng. 2019, 7, 9525–9536. 10.1021/acssuschemeng.9b01020. 31157127 PMC6537470

[ref36] Chung Y. G.; Haldoupis E.; Bucior B. J.; Haranczyk M.; Lee S.; Zhang H.; Vogiatzis K. D.; Milisavljevic M.; Ling S.; Camp J. S. Advances, Updates, and Analytics for the Computation-ready, Experimental Metal–Organic Framework Database: CoRE MOF 2019. J. Chem. Eng. Data 2019, 64, 5985–5998. 10.1021/acs.jced.9b00835.

[ref37] Frenkel D.; Smit B. From Algorithms to Applications, 2nd Ed.; Academic Press: San Diego, 2002; Vol. 1.

[ref38] Dubbeldam D.; Calero S.; Ellis D. E.; Snurr R. Q. RASPA: Molecular Simulation Software for Adsorption and Diffusion in Flexible Nanoporous Materials. Mol. Simul. 2016, 42, 81–101. 10.1080/08927022.2015.1010082.

[ref39] Rappé A. K.; Casewit C. J.; Colwell K.; Goddard W. III; Skiff W. UFF, A Full Periodic Table Force Field for Molecular Mechanics and Molecular Dynamics Simulations. J. Am. Chem. Soc. 1992, 114, 10024–10035. 10.1021/ja00051a040.

[ref40] Martin M. G.; Siepmann J. I. Transferable Potentials for Phase Equilibria. 1. United-Atom Description of n-Alkanes. J. Phys. Chem. B 1998, 102, 2569–2577. 10.1021/jp972543+.

[ref41] Buch V. Path Integral Simulations of Mixed Para-D_2_ and Ortho-D_2_ Clusters: The Orientational effects. J. Chem. Phys. 1994, 100, 7610–7629. 10.1063/1.466854.

[ref42] Makrodimitris K.; Papadopoulos G. K.; Theodorou D. N. Prediction of Permeation Properties of CO_2_ and N_2_ through Silicalite via Molecular Simulations. J. Phys. Chem. B 2001, 105, 777–788. 10.1021/jp002866x.

[ref43] Wilmer C. E.; Snurr R. Q. Towards Rapid Computational Screening of Metal-Organic Frameworks for Carbon Dioxide Capture: Calculation of Framework Charges via Charge Equilibration. Chem. Eng. J. 2011, 171, 775–781. 10.1016/j.cej.2010.10.035.

[ref44] Ewald P. P. Die Berechnung Optischer und Elektrostatischer Gitterpotentiale. Ann. Phys. 1921, 369, 253–287. 10.1002/andp.19213690304.

[ref45] Keskin S.; Sholl D. S. Assessment of a Metal–Organic Framework Membrane for Gas Separations Using Atomically Detailed Calculations: CO_2_, CH_4_, N_2_, H_2_ Mixtures in MOF-5. Ind. Eng. Chem. Res. 2009, 48, 914–922. 10.1021/ie8010885.

[ref46] Robeson L. M. The Upper Bound Revisited. J. Membr. Sci. 2008, 320, 390–400. 10.1016/j.memsci.2008.04.030.

[ref47] Maxwell J. C. A Treatise on Electricity and Magnetism; Dover Publications: New York, 1954; Vol. 2.

[ref48] Mukherjee K.; Colón Y. J. Machine Learning and Descriptor Selection for the Computational Discovery of Metal-organic Frameworks. Mol. Simul. 2021, 47, 1–21. 10.1080/08927022.2021.1916014.

[ref49] Altintas C.; Avci G.; Daglar H.; Gulcay E.; Erucar I.; Keskin S. Computer Simulations of 4240 MOF Membranes for H_2_/CH_4_ Separations: Insights Into Structure–Performance Relations. J. Mater. Chem. A 2018, 6, 5836–5847. 10.1039/C8TA01547C. PMC6003548 30009024

[ref50] Yan Y.; Shi Z.; Li H.; Li L.; Yang X.; Li S.; Liang H.; Qiao Z. Machine Learning and In-silico Screening of Metal–Organic Frameworks for O_2_/N_2_ Dynamic Adsorption and Separation. Chem. Eng. J. 2022, 427, 131604 10.1016/j.cej.2021.131604.

[ref51] Qiao Z.; Yan Y.; Tang Y.; Liang H.; Jiang J. Metal–Organic Frameworks for Xylene Separation: From Computational Screening to Machine Learning. J. Phys. Chem. C 2021, 125, 7839–7848. 10.1021/acs.jpcc.0c10773.

[ref52] Liang H.; Jiang K.; Yan T.-A.; Chen G.-H. XGBoost: An Optimal Machine Learning Model with Just Structural Features to Discover MOF Adsorbents of Xe/Kr. ACS Omega 2021, 6, 9066–9076. 10.1021/acsomega.1c00100. 33842776 PMC8028164

[ref53] Shi Z.; Yuan X.; Yan Y.; Tang Y.; Li J.; Liang H.; Tong L.; Qiao Z. Techno-Economic Analysis of Metal–Organic Frameworks for Adsorption Heat Pumps/Chillers: from Directional Computational Screening, Machine Learning to Experiment. J. Mater. Chem. A 2021, 9, 7656–7666. 10.1039/D0TA11747A.

[ref54] Pardakhti M.; Nanda P.; Srivastava R. Impact of Chemical Features on Methane Adsorption by Porous Materials at Varying Pressures. J. Phys. Chem. C 2020, 124, 4534–4544. 10.1021/acs.jpcc.9b09319.

[ref55] Willems T. F.; Rycroft C. H.; Kazi M.; Meza J. C.; Haranczyk M. Algorithms and Tools for High-throughput Geometry-based Analysis of Crystalline Porous Materials. Microporous Mesoporous Mater. 2012, 149, 134–141. 10.1016/j.micromeso.2011.08.020.

[ref56] Yang P.; Zhang H.; Lai X.; Wang K.; Yang Q.; Yu D. Accelerating the Selection of Covalent Organic Frameworks with Automated Machine Learning. ACS Omega 2021, 6, 17149–17161. 10.1021/acsomega.0c05990. 34278102 PMC8280634

[ref57] Olson R. S.; Urbanowicz R. J.; Andrews P. C.; Lavender N. A.; Kidd L. C.; Moore J. H. Automating Biomedical Data Science Through Tree-based Pipeline Optimization. Applications of Evolutionary Computation; Springer, 2016; pp 123–137.

[ref58] Yao Q.; Wang M.; Chen Y.; Dai W.; Li Y.-F.; Tu W.-W.; Yang Q.; Yu Y. Taking Human out of Learning Applications: A Survey on Automated Machine Learning. arXiv preprint arXiv:1810.13306. arXiv.org e-Print archive, 2018. https://doi.org/10.48550/arXiv.1810.13306.

[ref59] Martinsson P.-G.; Rokhlin V.; Tygert M. A Randomized Algorithm for the Decomposition of Matrices. Appl. Comput. Harmon. Anal. 2011, 30, 47–68. 10.1016/j.acha.2010.02.003.

[ref60] Wilmer C. E.; Leaf M.; Lee C. Y.; Farha O. K.; Hauser B. G.; Hupp J. T.; Snurr R. Q. Large-scale Screening of Hypothetical Metal–organic Frameworks. Nat. Chem. 2012, 4, 83–89. 10.1038/nchem.1192. 22270624

[ref61] Yan Y.; Shi Z.; Li H.; Lifeng L.; Yang X.; Li S.; Liang H.; Qiao Z. Machine Learning and In-silico Screening of Metal–Organic Frameworks for O_2_/N_2_ Dynamic Adsorption and Separation. Chem. Eng. J. 2022, 427, 131604 10.1016/j.cej.2021.131604.

[ref62] Mao Y.; Cao W.; Li J.; Liu Y.; Ying Y.; Sun L.; Peng X. Enhanced Gas Separation through Well-intergrown MOF Membranes: Seed Morphology and Crystal Growth Effects. J. Mater. Chem. A 2013, 1, 11711–11716. 10.1039/c3ta12402a.

[ref63] Cao F.; Zhang C.; Xiao Y.; Huang H.; Zhang W.; Liu D.; Zhong C.; Yang Q.; Yang Z.; Lu X. Helium Recovery by a Cu-BTC Metal–Organic-Framework Membrane. Ind. Eng. Chem. Res. 2012, 51, 11274–11278. 10.1021/ie301445p.

[ref64] Nan J.; Dong X.; Wang W.; Jin W. Formation Mechanism of Metal–organic Framework Membranes Derived from Reactive Seeding Approach. Microporous Mesoporous Mater. 2012, 155, 90–98. 10.1016/j.micromeso.2012.01.010.

[ref65] Akbari A.; Karimi-Sabet J.; Ghoreishi S. M. Matrimid 5218 based Mixed Matrix Membranes Containing Metal Organic Frameworks (MOFs) for Helium Separation. Chem. Eng. Process. 2020, 148, 107804 10.1016/j.cep.2020.107804.

[ref66] Hsieh J. O.; Balkus K. J. Jr; Ferraris J. P.; Musselman I. H. MIL-53 Frameworks in Mixed-Matrix Membranes. Microporous Mesoporous Mater. 2014, 196, 165–174. 10.1016/j.micromeso.2014.05.006.

[ref67] Aliyev E.; Warfsmann J.; Tokay B.; Shishatskiy S.; Lee Y.-J.; Lillepaerg J.; Champness N. R.; Filiz V. Gas Transport Properties of the Metal–organic Framework (MOF)-assisted Polymer of Intrinsic Microporosity (PIM-1) Thin-film Composite Membranes. ACS Sustainable Chem. Eng. 2020, 9, 684–694. 10.1021/acssuschemeng.0c06297.

[ref68] Zhang H.; Nettleton D.; Zhu Z. Regression-Enhanced Random Forests. arXiv preprint arXiv:1904.10416, arXiv.org e-Print archive, 2019. https://doi.org/10.48550/arXiv.1904.10416.

