# Supplementary material for: Combining Machine Learning and Molecular Simulations to Unlock Gas Separation Potentials of MOF Membranes and MOF/Polymer MMMs
Source: ACS Appl Mater Interfaces. 2022 Jul 12;14(28):32134–48. doi: 10.1021/acsami.2c08977 (PMC9305976; doi:10.1021/acsami.2c08977)
Supplement: Supplementary file 1 — am2c08977_si_001.pdf [file am2c08977_si_001.pdf]

Supporting Information

Combining Machine Learning and Molecular Simulations to Unlock Gas Separation Potentials of MOF Membranes and MOF/Polymer MMMs

Hilal Daglar[a] and Seda Keskin[a*]

[a]Department of Chemical and Biological Engineering, Koc University, Rumelifeneri Yolu, Sariyer, 34450, Istanbul, Turkey

Corresponding author: e-mail: skeskin@ku.edu.tr, Phone: +90 (212) 338-1362

Table S1. Cutoff thresholds.

Gas Properties	Upper Limit	Lower Limit
N_{He} (mol/kg)	1.55×10^{-1}	1.02×10^{-2}
N_{H_2} (mol/kg)	2.16×10^{-1}	1.00×10^{-2}
N_{CH_4} (mol/kg)	3.39	2.19×10^{-2}
N_{N_2} (mol/kg)	3.40	3.00×10^{-2}
D_{He} (cm^2/s)	4.38×10^{-3}	1.36×10^{-4}
D_{H_2} (cm^2/s)	5.40×10^{-3}	9.07×10^{-5}
D_{CH_4} (cm^2/s)	1.00×10^{-3}	2.75×10^{-7}
D_{N_2} (cm^2/s)	1.47×10^{-3}	9.47×10^{-7}

Table S2. The potential parameters of gas models.

Atoms	Charge (e^-)	ε/k^B (K)	σ (Å)
H_2	0.0	34.2	2.96
He	0.0	10.9	2.64
CH_4	0.0	148.0	3.73
O (O_2)	-0.113	49.0	3.02
Dummy (O_2)	0.226	-	-
N (N_2)	-0.405	38.298	3.31
Dummy (N_2)	0.810	-	-

Table S3. Gas permeability of pure polymers.

Polymers	Gas Permeability (Barrer)				Ref.
	He	H_2	N_2	CH_4	
Polypropylene	22.5	-	0.03617	0.00739	1
PBOI-2-Cu^+	-	3.7	0.00385417	-	2
HyflonAD60X	405	140	8.3	2.4	3
PTMSP-60/40	-	-	153	80.5	4
TeflonAF-2400	3600	3396	790	600	5
PTMSP	-	23,200	9350	23,317	4

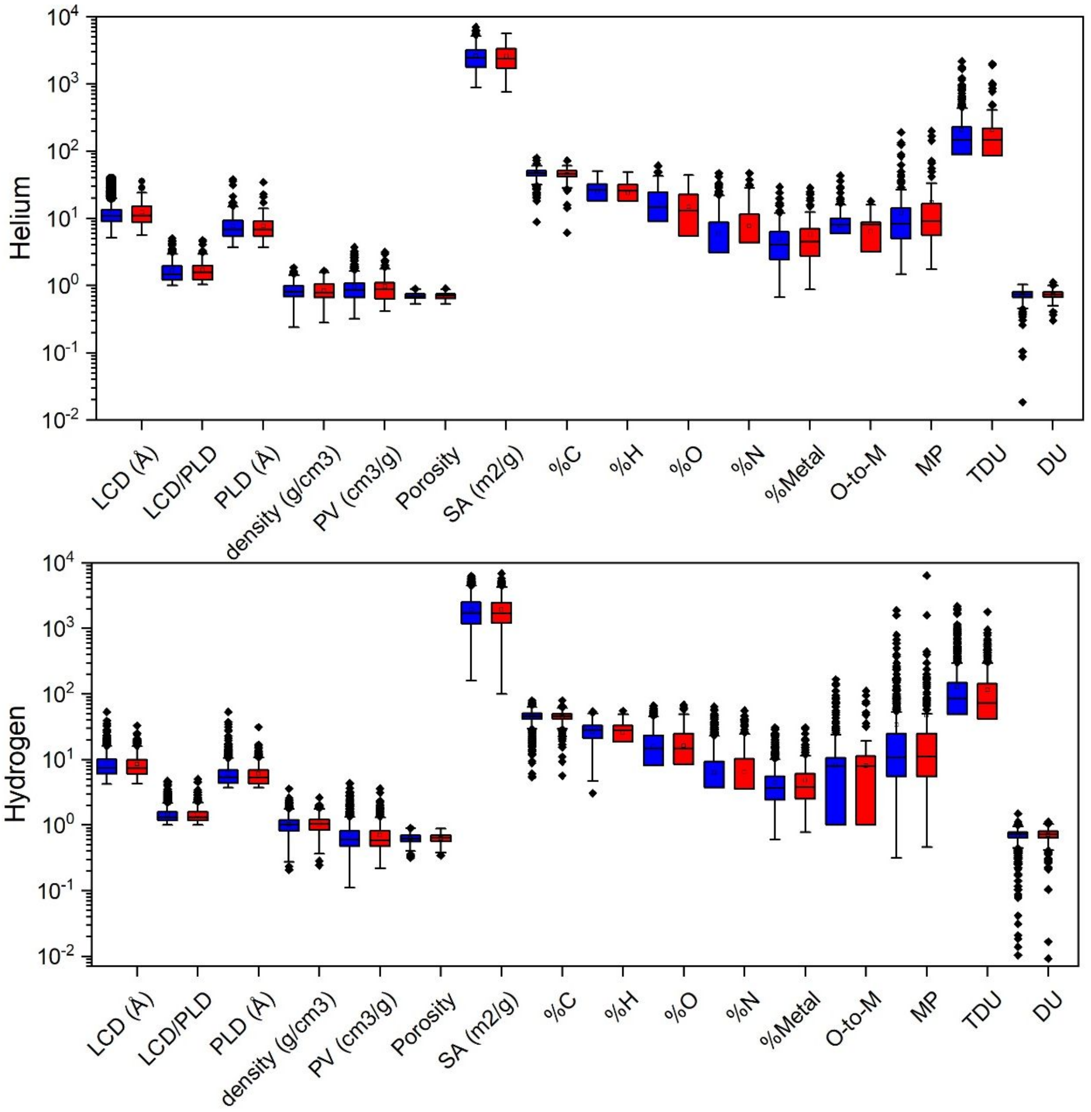

Figure S1. Box plots showing the distribution of features based on the training and test set for He and H_2. Blue (red) represents the training (test) set. Boxes show the quartiles of the dataset, while whiskers extend to show the rest of the distribution, except for the outliers which were defined as values more than 1.5IQR (IQR = interquartile range) from either end of the box.

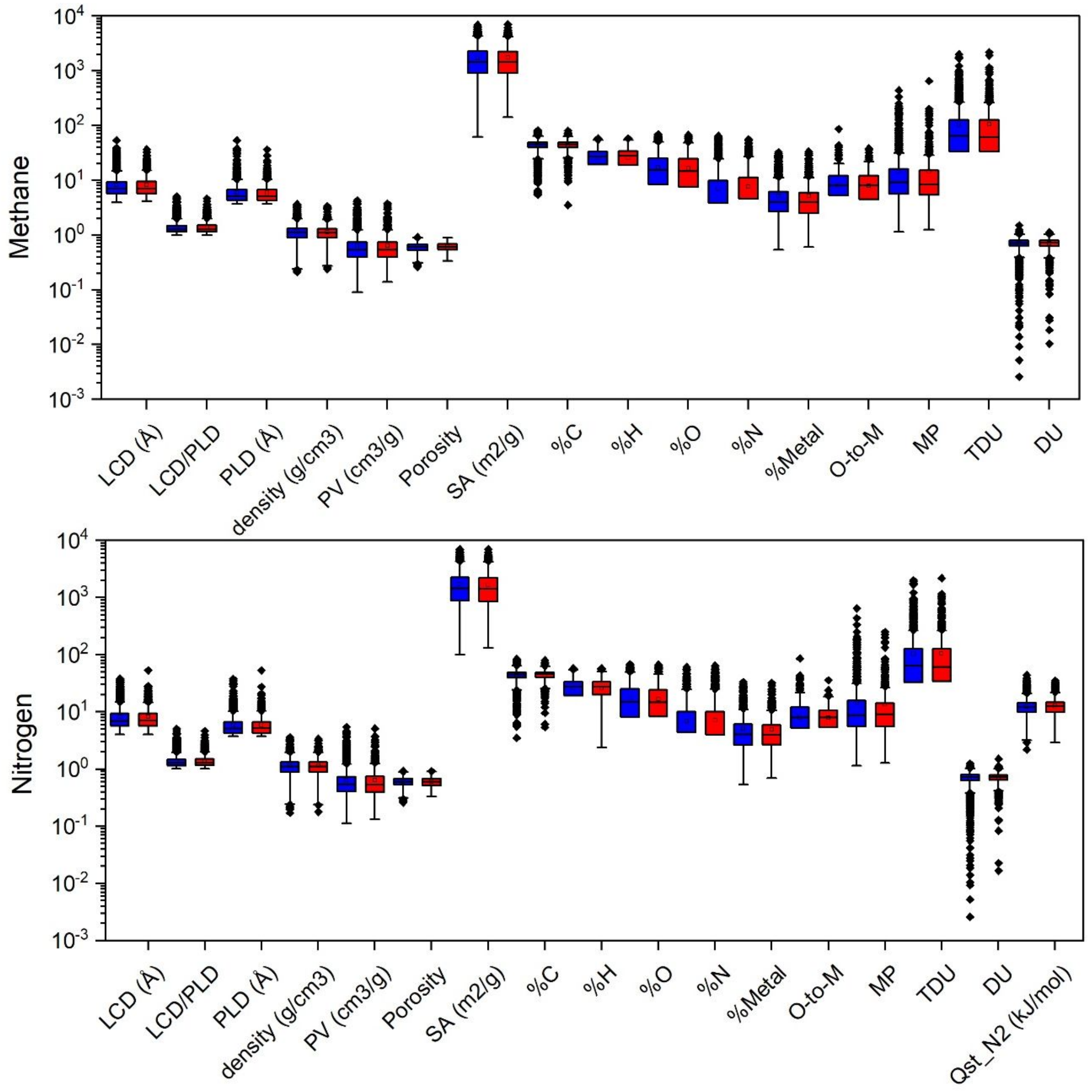

Figure S2. Box plots showing the distribution of features based on the training and test set for CH_4 and N_2. Blue (red) represents the training (test) set. Boxes show the quartiles of the dataset, while whiskers extend to show the rest of the distribution, except for the outliers which were defined as values more than 1.5IQR (IQR = interquartile range) from either end of the box.

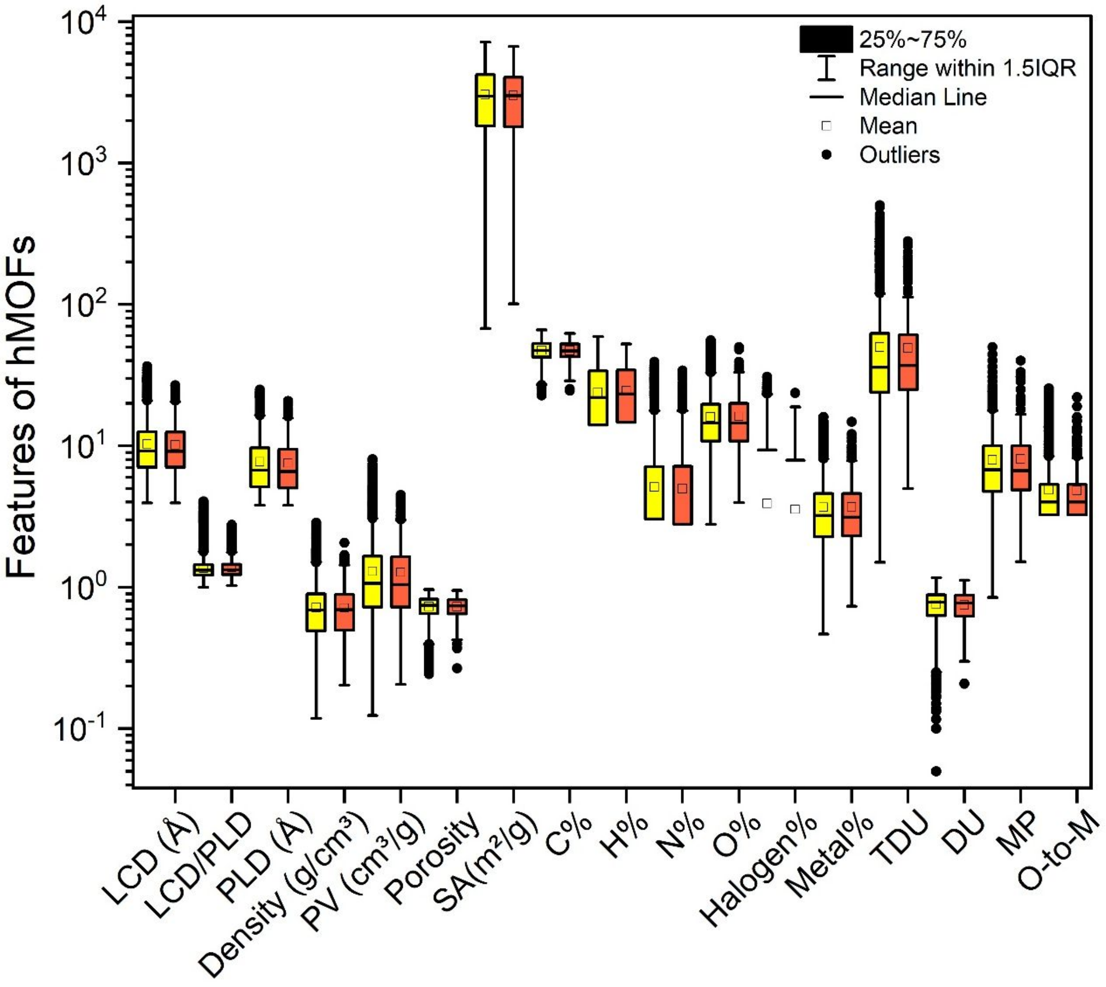

Figure S3. Box plots showing the distribution of features based on hMOFs. Yellow and orange symbols represent the curated hMOF set including 102,926 materials and representative hMOF set including 500 materials. Boxes show the quartiles of the dataset, while whiskers extend to show the rest of the distribution, except for points that are determined to be outliers.

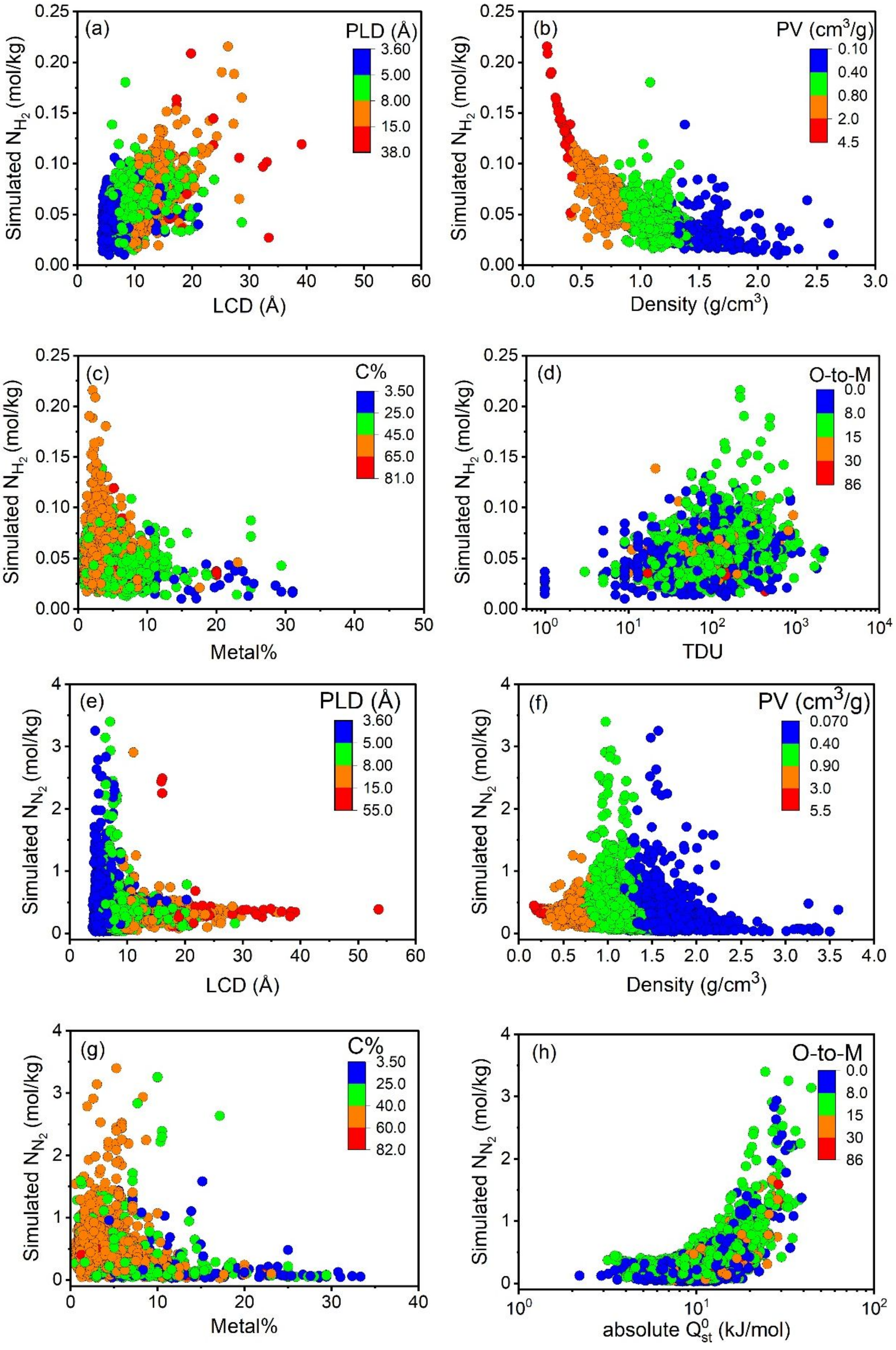

Figure S4. The effect of features on gas adsorption: Simulated H_2 and N_2 uptakes of 2715 and 5224 MOFs, respectively, as a function of pore size (LCD, PLD), pore geometry (density, pore volume), atom types (C%, metal%), chemical descriptors (O-to-M, TDU) and energy descriptor (Q_{st}) in N_2 case.

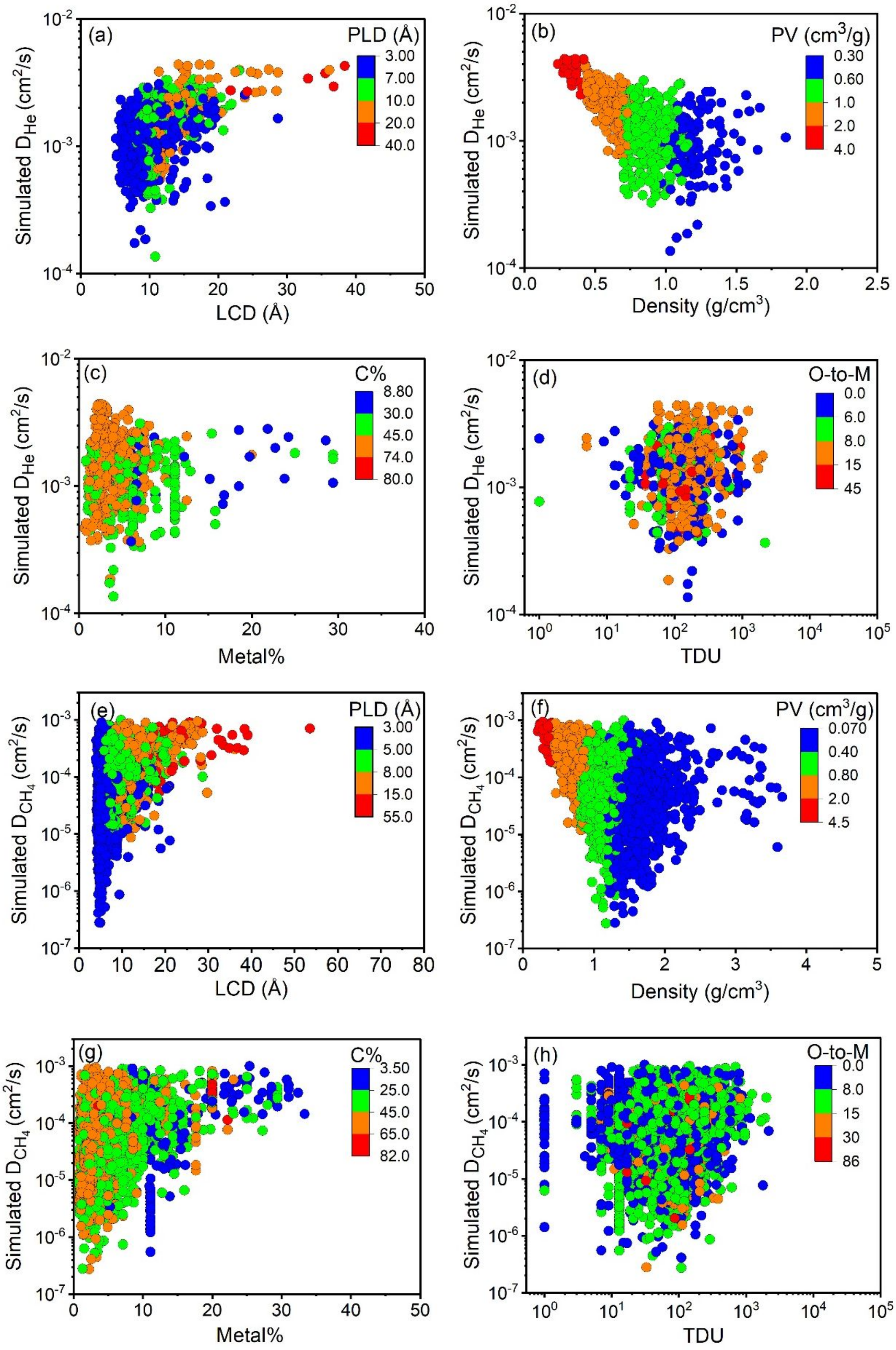

Figure S5. The effect of features on gas diffusion: Simulated He and CH_4 diffusivities in 677 and 5215 MOFs, respectively, as a function of pore size (LCD, PLD), pore geometry (density, pore volume), atom types (C%, metal%) and chemical descriptors (O-to-M, TDU).

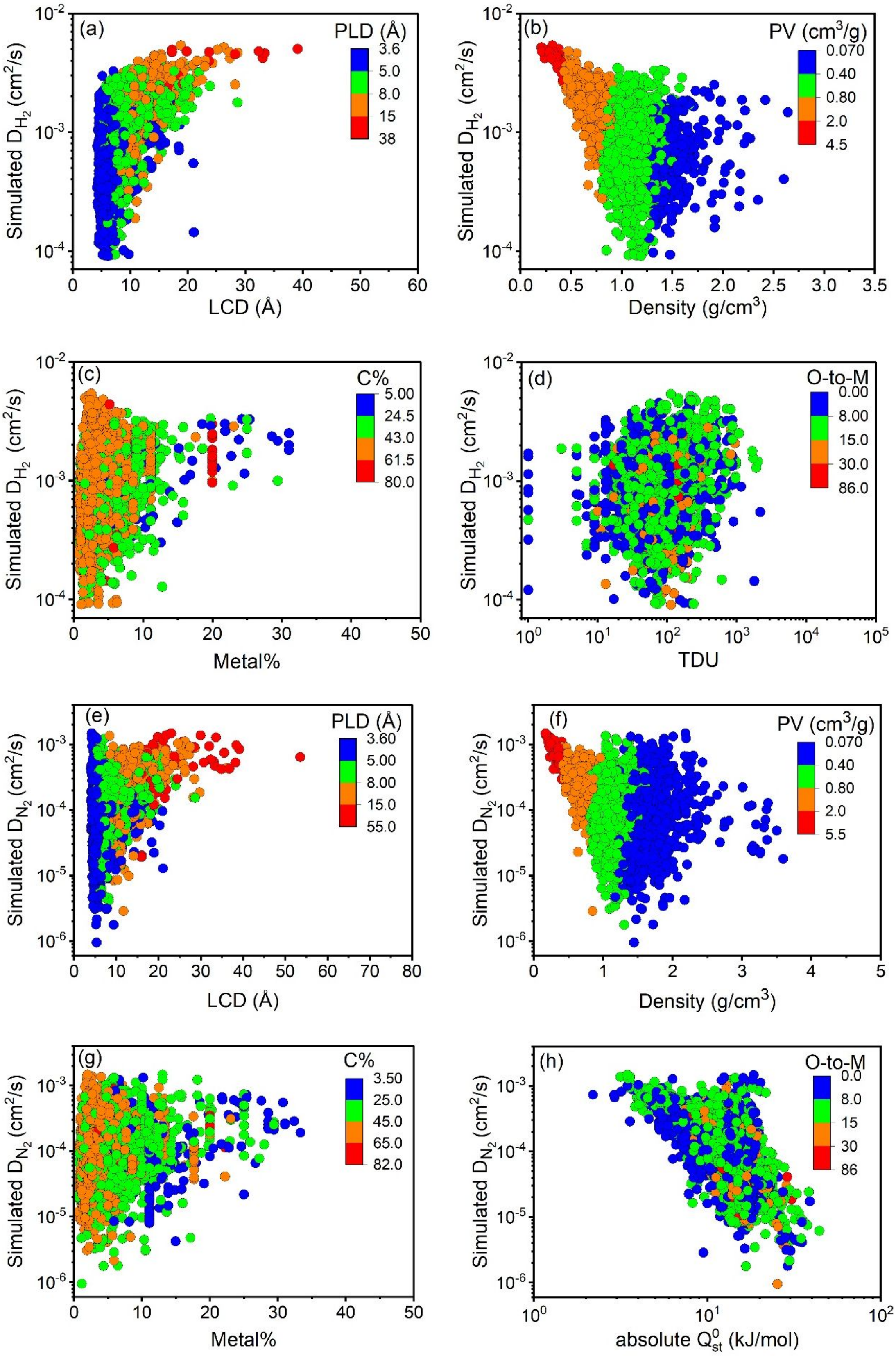

Figure S6. The effect of features on gas diffusion: Simulated H_2 and N_2 diffusivities in 2715 and 5224 MOFs, respectively, as a function of pore size (LCD, PLD), pore geometry (density, pore volume), atom types (C%, metal%), chemical descriptors (O-to-M, TDU) and energy descriptor (Q_{st}) in N_2 case.

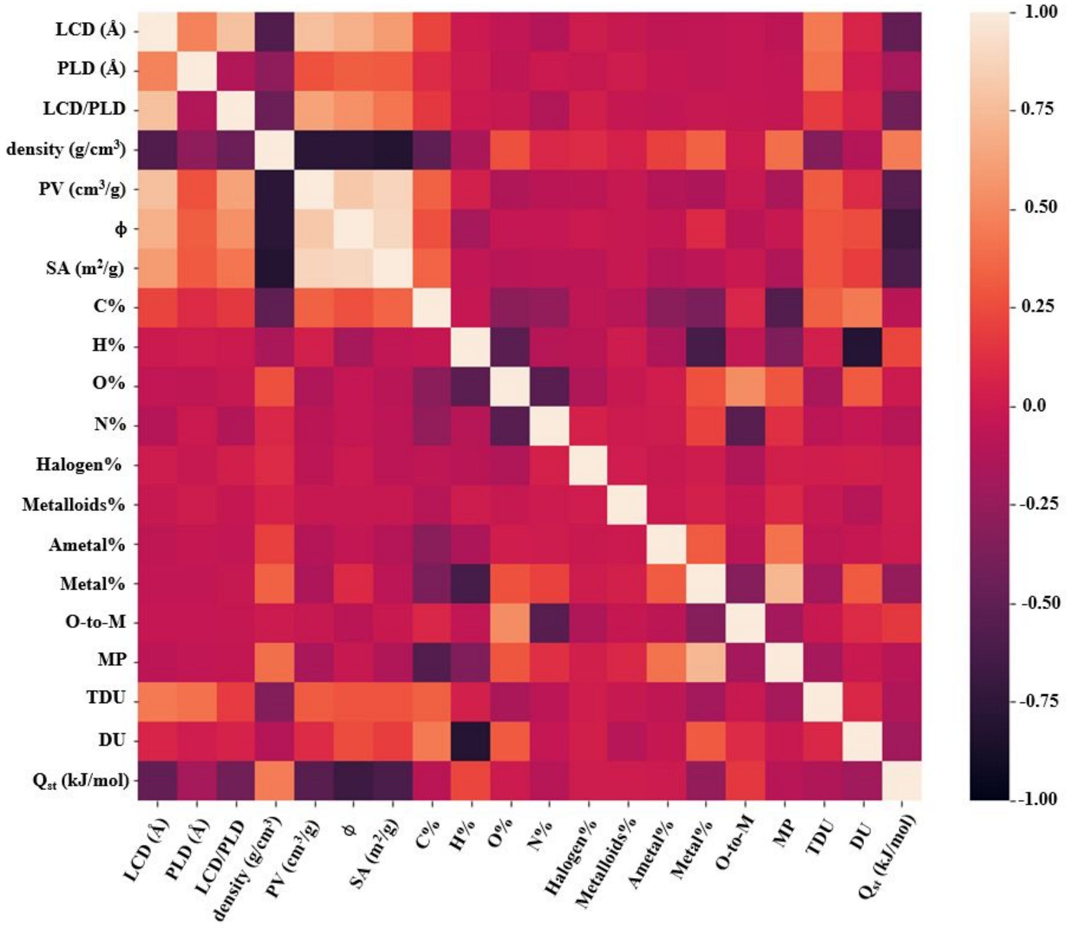

Figure S7. Correlation heat map of physical and chemical and energetic features of MOFs.

Table S4. The ML pipelines and parameters based on gas adsorption and diffusion properties.

**Property**	**Best Pipeline with Parameters**
He adsorption	**LassoLarsCV** (PolynomialFeatures(degree=2, include_bias=False, interaction_only=False), normalize=False))
H_2 adsorption	**ExtraTreesRegressor** (MaxAbsScaler(), bootstrap=False, max_features=0.55, min_samples_leaf=2, min_samples_split=5, n_estimators=100)
N_2 adsorption	**GradientBoostingRegressor** (StackingEstimator(estimator=RidgeCV()), (alpha=0.95, learning_rate=0.1, loss="ls", max_depth=10, max_features=0.1, min_samples_leaf=6, min_samples_split=12, n_estimators=100, subsample=0.9000000000000001))
CH_4 adsorption	**ExtraTreesRegressor** (PolynomialFeatures(degree=2, include_bias=False, interaction_only=False), bootstrap=False, max_features=0.2, min_samples_leaf=2, min_samples_split=5, n_estimators=100)
He diffusion	**ExtraTreesRegressor** (PolynomialFeatures(degree=2, include_bias=False, interaction_only=False), bootstrap=False, max_features=0.6000000000000001, min_samples_leaf=9, min_samples_split=19, n_estimators=100)
H_2 diffusion	**ExtraTreesRegressor** (bootstrap=False, max_features=0.55, min_samples_leaf=2, min_samples_split=5, n_estimators=100)
N_2 diffusion	**GradientBoostingRegressor** (alpha=0.95, learning_rate=0.1, loss="ls", max_depth=10, max_features=0.1, min_samples_leaf=6, min_samples_split=12, n_estimators=100, subsample=0.9000000000000001)
CH_4 diffusion	**RandomForestRegressor** (bootstrap=False, max_features=0.3, min_samples_leaf=3, min_samples_split=6, n_estimators=100)

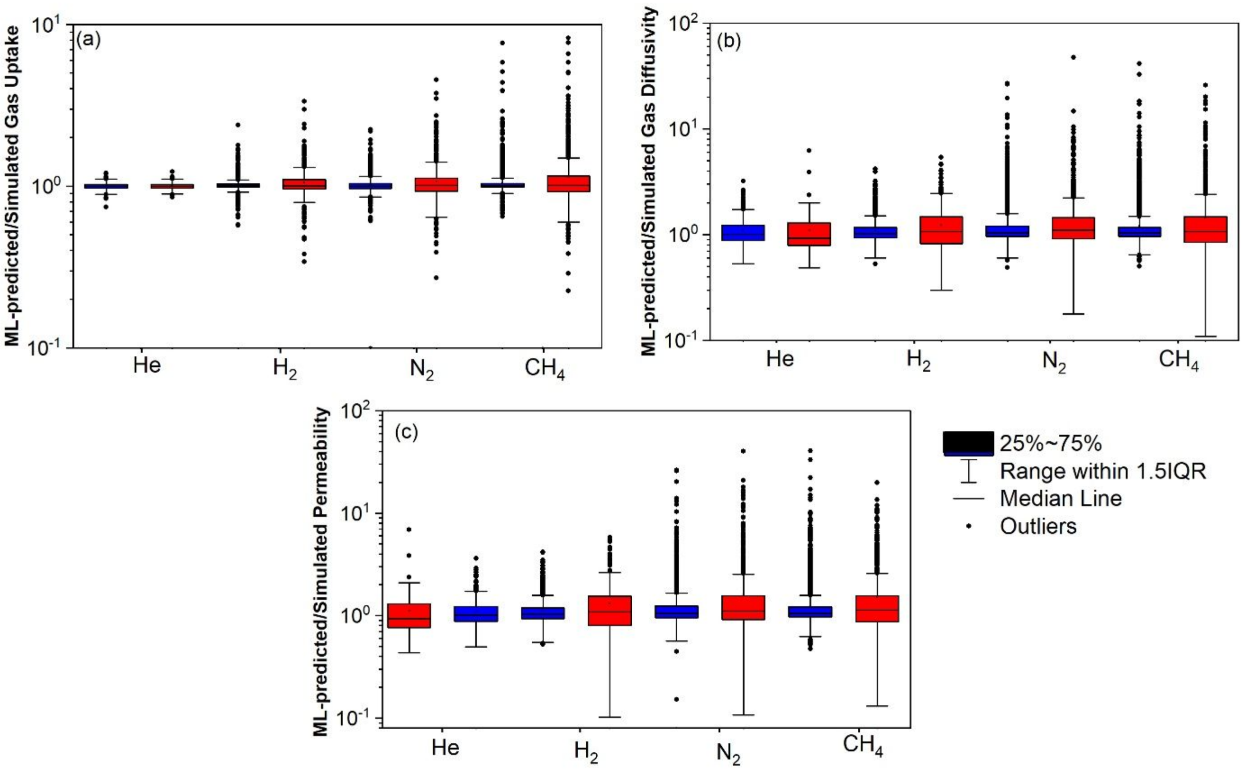

Figure S8. The ratio of ML-predicted uptake, diffusivity, and permeability to the simulated ones for He, H_2, N_2, and CH_4. Blue and red boxes represent the training and test set for each gas. Boxes show the quartiles of the dataset, while whiskers extend to show the rest of the distribution, except for points that are determined to be outliers. Outliers are defined as values more than 1.5IQR (IQR = interquartile range) from either end of the box.

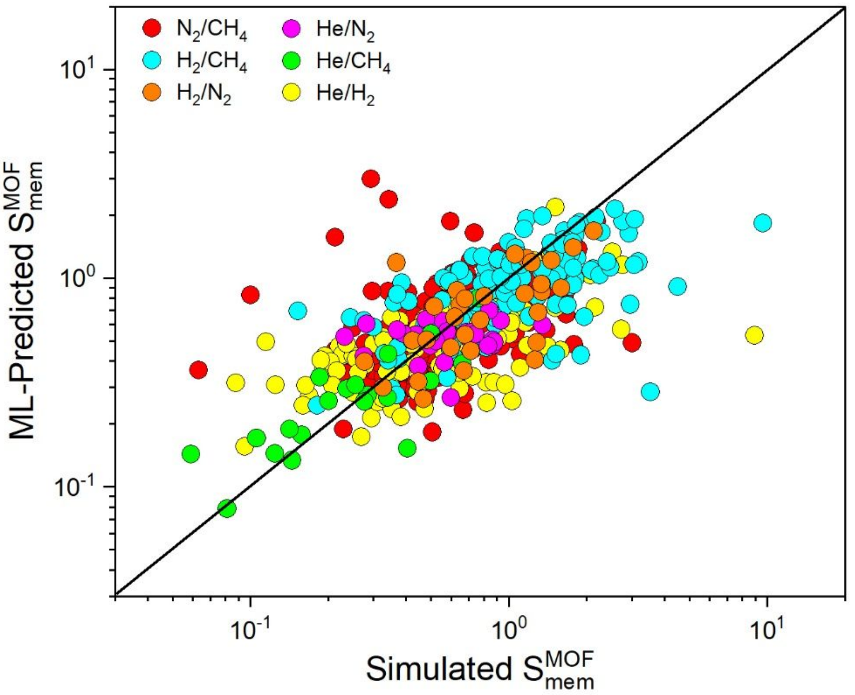

Figure S9. Comparison between the ML-predicted and simulated membrane selectivity of MOFs in the test set for He/H_2, He/N_2, He/CH_4, H_2/CH_4, H_2/N_2, N_2/CH_4 separations.

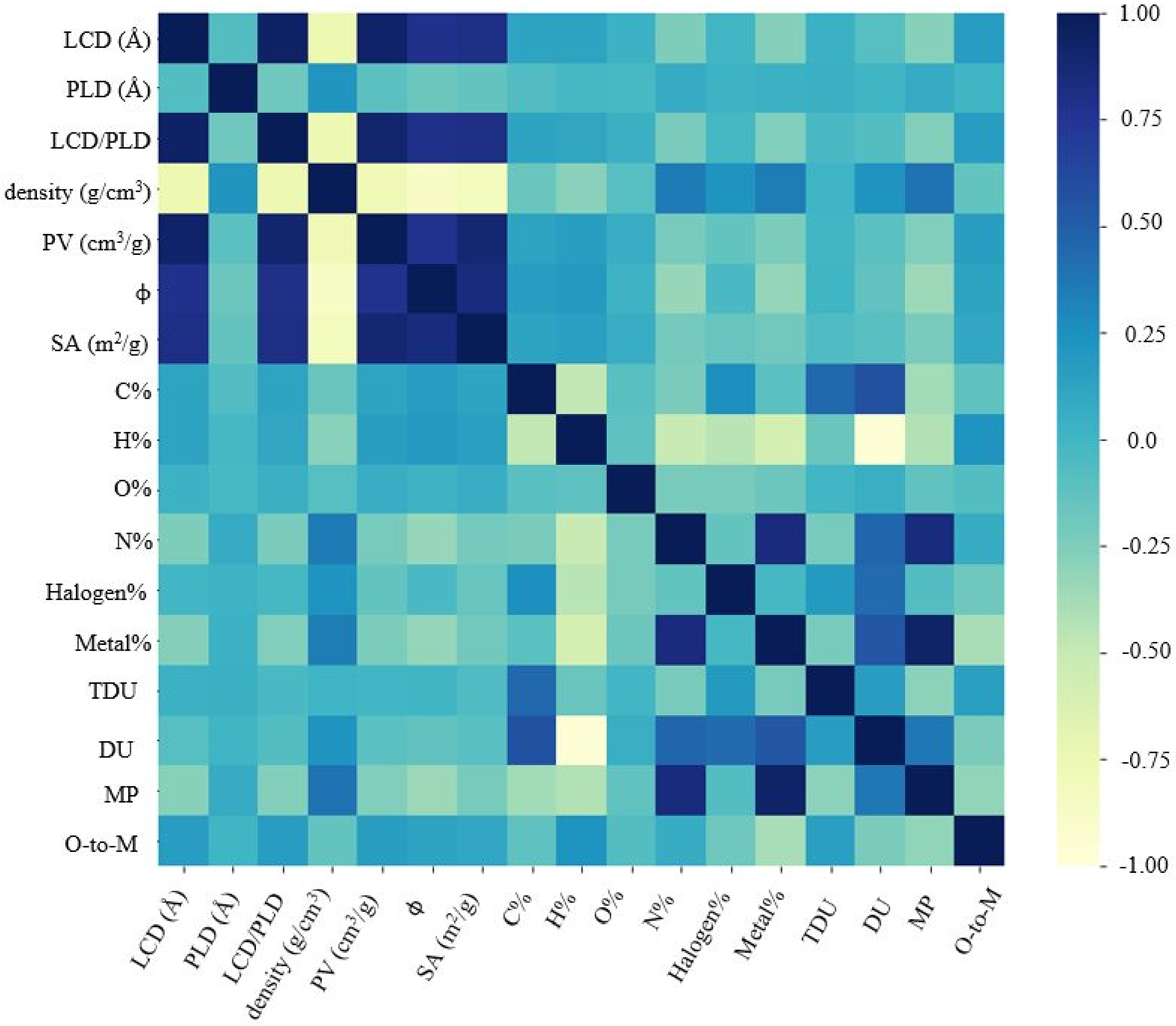

Figure S10. Correlation heatmap of the descriptors of hMOFs. Since there are no Metalloids (As, B, Ge, Te, Sb, Si) and Ametal (Se, S, P) groups in hMOFs, figure does not show columns and row corresponding to these atom types.

Table S5. MAE, RMSE, SRCC and R^2 of ML-predicted gas uptake and diffusivity in hMOFs in Figure 9(a-d).

hMOFs	RMSE	MAE	SRCC	R^2
He Uptake	2.34×10^{-3}	1.80×10^{-3}	0.997	0.997
He Diffusion	1.17×10^{-3}	6.48×10^{-4}	0.893	0.716
H_2 Uptake	8.02×10^{-3}	6.24×10^{-3}	0.946	0.940
H_2 Diffusion	1.08×10^{-3}	6.17×10^{-4}	0.897	0.738

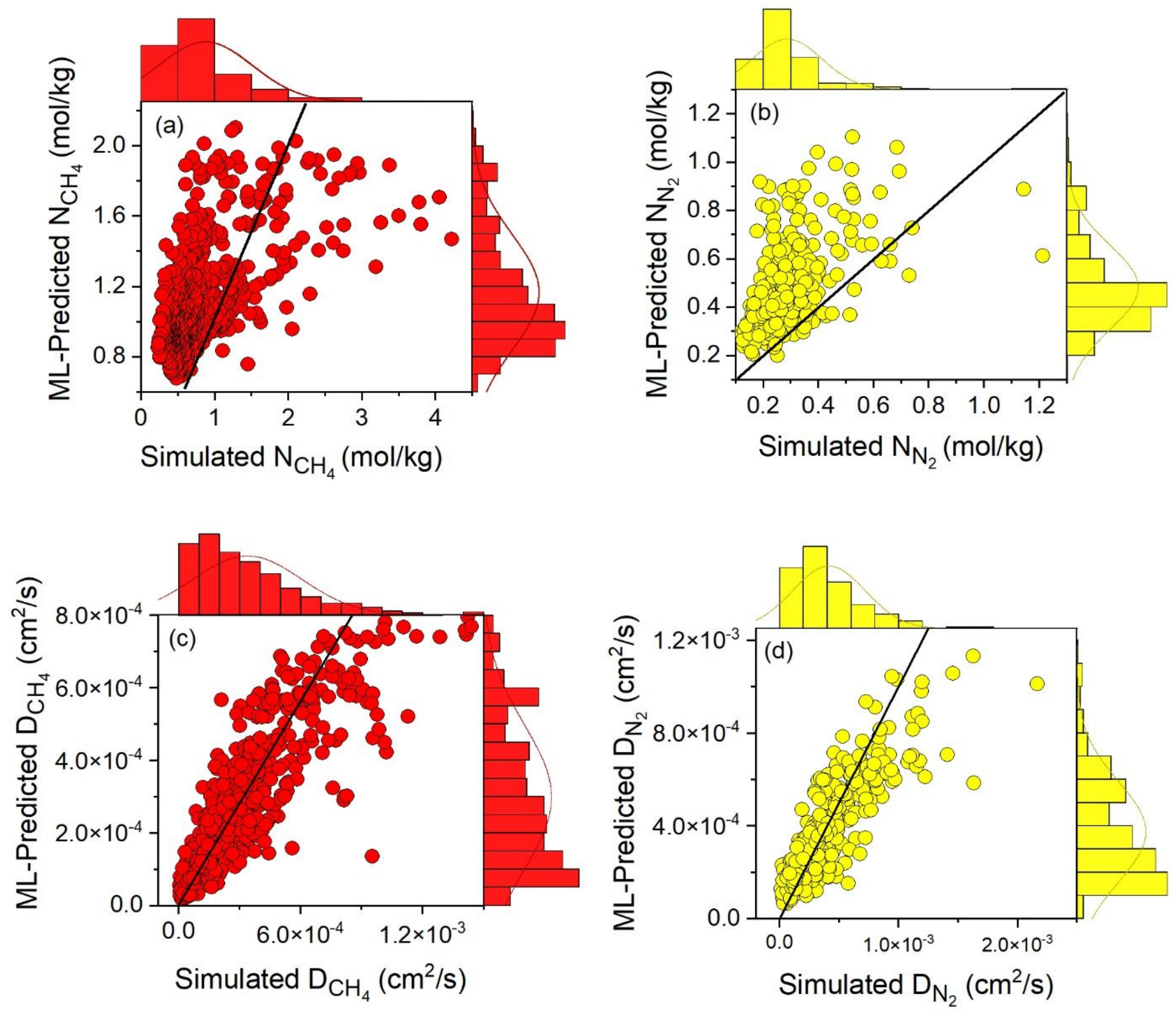

Figure S11. The comparison of the ML-predicted (a-b) uptake and (c-d) diffusivity for CH_4 and N_2 gases of unseen 500 hMOFs with the simulated ones. In N_2 case (b, d), we focused on the 350 hMOFs for which partial atomic charges of elements in the framework were assigned to be in the range of -0.5 and 2.

References

(1) Zimmerman, C.M.; Koros, W.J. Polypyrrolones for membrane gas separations. I. Structural Comparison of Gas Transport and Sorption Properties, J. Polym. Sci. B Polym. Phys., **1999**, *37*, 1235-1249.

(2) Polotskaya, G.; Goikhman, M.; Podeshvo, I.; Kudryavtsev, V.; Pientka, Z.; Brozova, L.; Bleha, M. Gas Transport Properties of Polybenzoxazinoneimides and Their Prepolymers, Polymer, **2005**, *46*, 3730-3736.

(3) Macchione, M.; Jansen, J.C.; De Luca, G.; Tocci, E.; Longeri, M.; Drioli, E. Experimental Analysis and Simulation of the Gas Transport in Dense Hyflon® AD60X Membranes: Influence of Residual Solvent, Polymer, **2007**, *48*, 2619-2635.

(4) Nagai, K.; Higuchi, A.; Nakagawa, T. Gas Permeability and Stability of Poly (1-trimethylsilyl-1-propyne-co-1-phenyl-1-propyne) Membranes, J. Polym. Sci. B Polym. Phys., **1995**, *33*, 289-298.

(5) Pinnau, I.; Toy, L.G. Gas and Vapor Transport Properties of Amorphous Perfluorinated Copolymer Membranes based on 2, 2-bistrifluoromethyl-4, 5-difluoro-1, 3-dioxole/tetrafluoroethylene, J. Membr. Sci., **1996**, *109*, 125-133.
